# BAP1 enhances Polycomb repression by counteracting widespread H2AK119ub1 deposition and chromatin condensation

**DOI:** 10.1016/j.molcel.2021.06.020

**Published:** 2021-09-02

**Authors:** Eric Conway, Federico Rossi, Daniel Fernandez-Perez, Eleonora Ponzo, Karin Johanna Ferrari, Marika Zanotti, Daria Manganaro, Simona Rodighiero, Simone Tamburri, Diego Pasini

**Affiliations:** 1 IEO, European Institute of Oncology IRCCS, Department of Experimental Oncology, Via Adamello 16, 20139 Milan, Italy; 2 University of Milan, Via A. di Rudini 8, Department of Health Sciences, 20142 Milan, Italy

**Keywords:** Polycomb, BAP1, PR-DUB, PRC1, PRC2, PCGF3, H2AK119ub1, transcriptional repression, chromatin compaction, tumor suppressor

## Abstract

BAP1 is mutated or deleted in many cancer types, including mesothelioma, uveal melanoma, and cholangiocarcinoma. It is the catalytic subunit of the PR-DUB complex, which removes PRC1-mediated H2AK119ub1, essential for maintaining transcriptional repression. However, the precise relationship between BAP1 and Polycombs remains elusive. Using embryonic stem cells, we show that BAP1 restricts H2AK119ub1 deposition to Polycomb target sites. This increases the stability of Polycomb with their targets and prevents diffuse accumulation of H2AK119ub1 and H3K27me3. Loss of BAP1 results in a broad increase in H2AK119ub1 levels that is primarily dependent on PCGF3/5-PRC1 complexes. This titrates PRC2 away from its targets and stimulates H3K27me3 accumulation across the genome, leading to a general chromatin compaction. This provides evidence for a unifying model that resolves the apparent contradiction between BAP1 catalytic activity and its role *in vivo*, uncovering molecular vulnerabilities that could be useful for BAP1-related pathologies.

## Highlights

• BAP1 prevents H2AK119ub1 accumulation across the genome, preserving Polycomb repression
• BAP1 loss accumulates H2AK119ub1 diffusely, inducing global compaction of chromatin
• Pathologic H2AK119ub1 accumulation is mainly sustained by the activity of PRC1.3/5
• PRC1.3/5 are attractive molecular targets for BAP1-null cancers

## Graphical abstract

**Figure:**
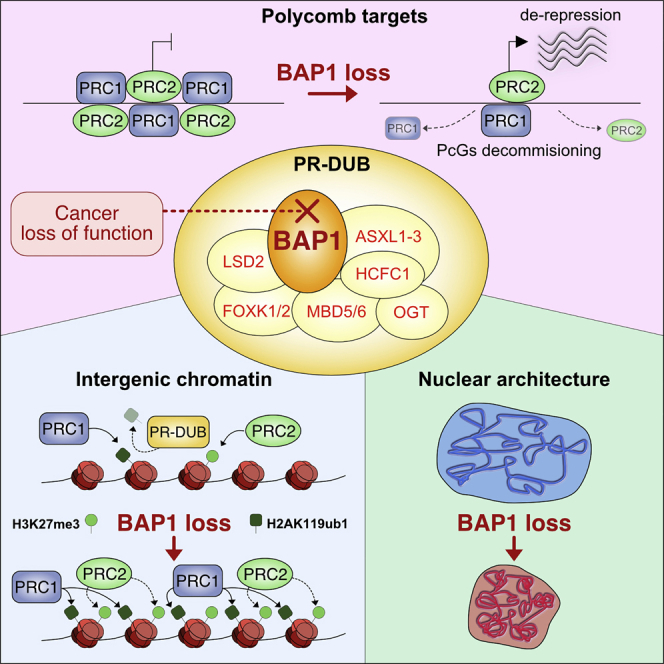

## Teaser

Conway et al. demonstrate that BAP1 sustains Polycomb-mediated repression by “bookending” chromatin. This occurs through the removal of diffuse genome-wide H2AK119ub1 deposition, enhancing Polycomb repression at target promoters and preventing aberrant chromatin compaction. This sheds light on BAP1 tumor-suppressive mechanisms and identifies new potential therapeutic strategies within PRC1 subcomplexes.

## Introduction

BAP1 is a ubiquitin C-terminal hydroxylase (UCH) with a substrate preference for histone H2A lysine 119 mono-ubiquitination (H2AK119ub1) (Sahtoe et al., 2016; Scheuermann et al., 2010). It is recurrently mutated or deleted with high frequency in mesothelioma (∼36%), uveal melanoma (∼40%), cholangiocarcinoma (∼26%), and renal clear cell carcinoma (∼24%), among others, where it functions as a tumor suppressor (Carbone et al., 2013; Cerami et al., 2012; Gao et al., 2013). BAP1 forms the Polycomb repressive de-ubiquitinase complex (PR-DUB), which contains the ASXL1-3 proteins along with other subunits, FOXK1/2, HCFC1, OGT, MBD5/6, and KDM1B (Hauri et al., 2016; Kloet et al., 2016). ASXL subunits are also mutated in myeloid cancers and neurodevelopmental disorders (Bohring-Opitz and Bainbridge-Ropers syndromes), linking the PR-DUB activity and H2AK119ub1 to disease pathogenesis (Bainbridge et al., 2013; Bejar et al., 2011; Gelsi-Boyer et al., 2009; Hoischen et al., 2011).

The precise BAP1 tumor-suppressive mechanism remains unclear. The DUB activity of BAP1 for H2AK119ub1 suggests an antagonistic relationship with the Polycomb repressive complex PRC1, which catalyzes H2AK119ub1 through the RING1A/B E3 ligases (Blackledge et al., 2015). PRC1 has vital roles in transcriptional repression, particularly during development and cell fate decisions (Deevy and Bracken, 2019). It is comprised of six major complex subtypes defined by the PCGF1-6 paralogs that form the catalytic core with RING1A/B (Gao et al., 2012). PRC1 sub-complexes can be broadly divided into canonical (PCGF2/4-containing) or variant (PCGF1/3/5/6-containing) groups, with specific genomic localizations and catalytic activities (Fursova et al., 2019; Scelfo et al., 2019). H2AK119ub1 is essential for repression of PRC1 target genes in embryonic stem cells (ESCs) and for the recruitment of PRC2 complexes (Blackledge et al., 2020; Tamburri et al., 2020). Moreover, H2AK119ub1 is required for Polycombrecruitment to the inactive X chromosome (Almeida et al., 2017), through a mechanism that involves the affinity of the PRC2 subunit JARID2 for H2AK119ub1 (Cooper et al., 2016).

Mutations of the BAP1 homolog *Calypso* cause a classical Polycomb anteriorization of *Hox* gene expression, due to a loss of repression (Scheuermann et al., 2010). Enzymatically, this remains counterintuitive, but it is further supported by studies in mice. While *Bap1* knockout is lethal around gastrulation (Dey et al., 2012), mutation of *Asxl1* and/or *Asxl2* displays both Trithorax and Polycomb transformations (Baskind et al., 2009; Fisher et al., 2010), suggesting a dual role in promoting and suppressing Polycomb activities. Transcriptional defects in *Bap1* knockout cells can be rescued by PRC1 deletion, although the mechanism behind this is unclear (Campagne et al., 2019). Some reports suggested that PR-DUB promotes PRC2 recruitment and that maintenance of H3K27me3 at target genes is dependent on ASXL proteins (Abdel-Wahab et al., 2012). As a result of this molecular ambiguity, it has been difficult to determine molecular sensitivities or synthetic lethalities for BAP1-mutated cancers (LaFave et al., 2015; Schoumacher et al., 2016).

Here we provide a unifying model for the role of BAP1 in promoting and limiting Polycomb complex activities. We show that, while PR-DUB and PRC1/2 share very few target genes, BAP1 activity is required for Polycomb occupancy at target sites. Loss of BAP1 causes spreading of H2AK119ub1 intergenically, which titrates away Polycomb complexes from their targets, in turn boosting intergenic H3K27me3 and depleting it at promoters. This facilitates activation of Polycomb target genes. In a mechanism reminiscent of X chromosome inactivation (XCI), the intergenic spreading of Polycomb modifications causes global chromatin compaction. Like XCI, this activity is dependent primarily on the PCGF3/5 PRC1 forms (Almeida et al., 2017; Fursova et al., 2019). Such dependency is preserved in BAP1 null cancer models addicted to hyper-H2AK119ub1 accumulation, making PCGF3/5-containing vPRC1 complexes attractive targets for these tumor types. This provides a model in which BAP1 is required to maintain local concentrations of PRC2 and H3K27me3 sufficient for transcriptional regulation while exposing potential therapeutic sensitivities for BAP1 mutant cancers.

## Results

### BAP1 binds active gene promoters and is excluded from Polycomb repressive domains

**Figure 1:**
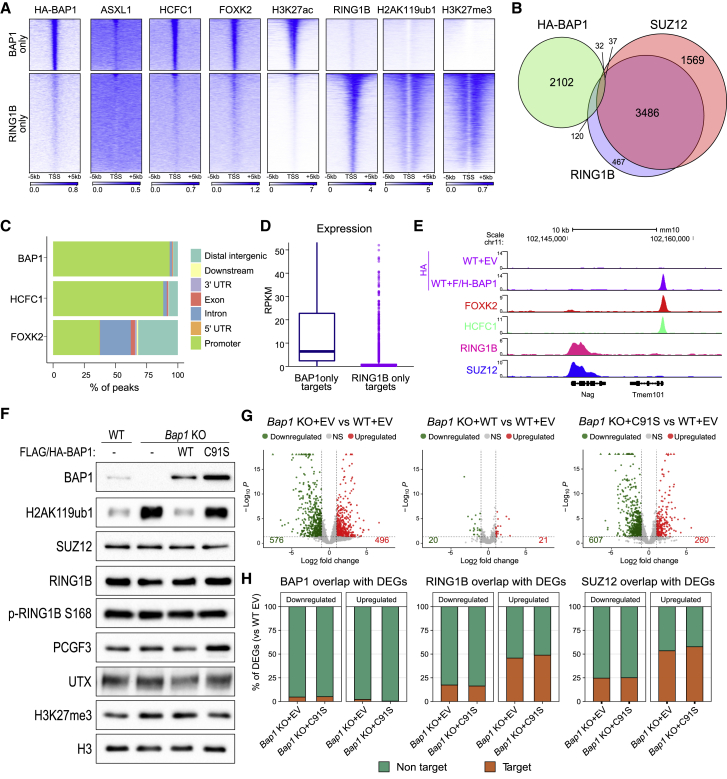
BAP1 binds active gene promoters and is excluded from Polycomb repressive domains (A) Heatmaps representing ChIP-seq intensity of the indicated proteins in wild-type ESCs. (B) Venn diagram of HA-BAP1, SUZ12, and RING1B target genes in ESCs. (C) Genome-wide functional annotation of peaks generated from the indicated ChIP-seq analyses. (D) Boxplots showing the expression levels obtained from RNA-seq analyses in WT mESCs for the clusters of target genes generated in (A). (E) Genome browser snapshot of ChIP-seq tracks showing an example of mutual exclusivity of PRC1/2 and PR-DUB target genes. (F) Western blot analysis with the indicated antibodies on total protein extracts from the indicated rescue ESC cell lines (E14 WT + empty vector, *Bap1* KO + empty vector, *Bap1* KO + BAP1 WT, *Bap1* KO + BAP1 C91S). (G) Volcano plots of −log10 (p value) against log2 fold change representing the differences in gene expression in the indicated cell lines. (H) Percentage overlap of differentially expressed genes (DEGs) from (G) with either HA-BAP1, RING1B, or SUZ12 ChIP-seq targets. See also Figure S1 and Tables S1, S2, and S3.

To investigate the relationship between PR-DUB, PRC1, and PRC2, we used mouse ESCs wherein Polycomb activities have been extensively characterized (Højfeldt et al., 2019; Scelfo et al., 2019; Tamburri et al., 2020). Since BAP1 genomic occupancy has been poorly understood until recently (Kolovos et al., 2020; Wang et al., 2018), with a dearth of commercially available ChIP-grade antibodies, we generated ESCs that stably expressed FLAG/HA tagged BAP1 (Figure S1A). We performed anti-HA ChIP-seq (Figure S1B) and identified 2,291 BAP1 target genes (Figures 1A and 1B; Table S1). In addition, we performed ChIP-seq for other PR-DUB complex members (ASXL1, HCFC1, and FOXK2), histone modifications (H3K27ac, H2AK119ub1, and H3K27me3), and the PRC1 complex (RING1B) (Figure 1A; Table S1). We found that BAP1 poorly overlapped with PRC1 and PRC2 (RING1B and SUZ12) and classified target genes into two distinct groups: BAP1-only and RING1B-only targets (Figures 1A and 1B). Importantly, all other PR-DUB subunits colocalized at BAP1-only target genes with no sign of enrichment at RING1B-bound loci (Figures 1A and S1C). Overall, these results underscore the validity of our BAP1 ChIP-seq and demonstrate that BAP1 is excluded, even under conditions of ectopic expression, from Polycomb target loci.

BAP1, HCFC1, and FOXK2 distribution revealed that in ESCs PR-DUB is primarily bound at promoters with a minor association to putative distal regulatory sites (Figure 1C), in agreement with a recent report (Kolovos et al., 2020). While HCFC1 followed a similar behavior, FOXK2 showed a broader distribution (Figure 1C) that likely involves enhancer occupancy within other chromatin regulatory complexes (Baymaz et al., 2014). Transcriptionally, while RING1B targets were largely inactive, PR-DUB targets were instead actively transcribed (Figures 1D and S1D). This is in accordance with the lack of H3K27me3 and H2AK119ub1 repressive histone post-translational modifications (PTMs) and with the accumulation of H3K27ac (Figure 1A). Together, these data illustrate that PR-DUB stable chromatin association at promoters is largely independent of PRC1 and PRC2 (Figure 1E) and is primarily found at active promoters.

We generated knockout ESCs to investigate the effects of BAP1 loss. Western blot and qRT-PCR analysis of two *Bap1* KO ESC lines showed the absence of BAP1 expression and a global increase in H2AK119ub1 deposition (Figures S1E and S1F), as expected (Campagne et al., 2019). To further delineate the structural functions of BAP1 from its catalytic role, we rescued *Bap1* KO ESCs with stable expression of WT or catalytic BAP1 mutant (C91S) (Figure S1F). Western blots in these models demonstrated that, while WT BAP1 expression restored H2AK119ub1 levels, the catalytic-inactive C91S mutant did not (Figure 1F). Importantly, this occurred in the absence of major changes in the levels of other PR-DUB subunits, of the Polycomb components SUZ12 and RING1B or the inhibited phosphorylated form of RING1B (Gao et al., 2014), and of the H3K27me3-specific histone demethylase UTX (Figures 1F and S1E). Finally, BAP1 binding profiles and PR-DUB stoichiometric composition were largely unaffected in the BAP1 catalytic mutant (Figures S1G, S1H, and S1I).

RNA-seq in this system identified hundreds of differentially expressed genes (DEGs) both up- and downregulated in the absence of either BAP1 or its catalytic activity (Figure 1G; Table S2). Critically, these DEGs were almost completely rescued upon re-expression of BAP1 WT, demonstrating that they are directly caused by loss of BAP1 catalytic activity. DEGs included a very small number of BAP1 direct targets (Figure 1H), suggesting that BAP1 activity does not serve to counteract repressive signals and is dispensable to sustain target expression under homeostatic conditions. However, the DEGs included a high number of Polycomb targets, with a significant enrichment among the upregulated group (Figure 1H). This suggests that, although BAP1 does not associate with Polycomb targets, its deubiquitinase activity plays a direct role in sustaining their repression.

To extend this analysis to a differentiation system, we stimulated ESCs with all-*trans* retinoic acid (ATRA) for 24 h and performed RNA-seq. This revealed similar trends to ESCs, with a large number of DEGs found in the absence of BAP1 or its catalytic activity (Figure S1J). PCA analysis and heatmaps of DEGs induced by the presence of ATRA confirmed that *Bap1* KO+EV mimics *Bap1* KO+C91S (Figures S1K and S1L). This highlights that the majority of transcriptional defects are caused by loss of BAP1 catalytic activity. Consistent with BAP1 and ASXL mutants in *Drosophila* and mouse (Baskind et al., 2009; Fisher et al., 2010; Scheuermann et al., 2010), upregulated genes were highly enriched for developmental terms with an overrepresentation of Polycomb targets (Figures S1K and S1M).

### BAP1 loss causes global increases in H2AK119ub1 and displacement of PRC1 from target loci

**Figure 2:**
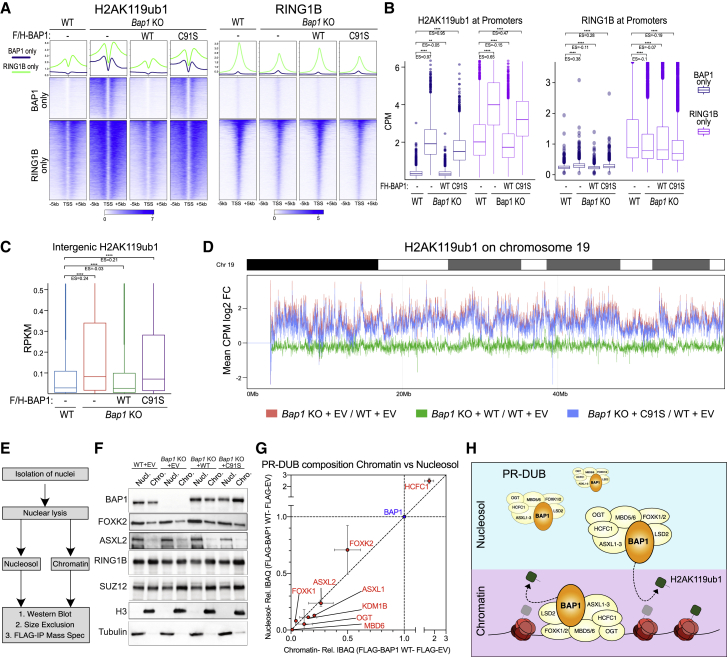
BAP1 loss causes global increases in H2AK119ub1 and displacement of PRC1 from target loci (A) Metaplots and heatmaps representing normalized ChIP-seq intensity for H2AK119ub1 or RING1B in the indicated cell lines. (B) Boxplot of normalized intensity profiles for H2AK119ub1 and RING1B ChIP-seq in the indicated cell lines. (C) Boxplot representing H2AK119ub1 ChIP-seq RPKM levels in the indicated cell lines at intergenic sites (n = 38,068). (D) Representation of the log2 fold change CPM in H2AK119ub1 ChIP-seq signal in the indicated cell lines across chromosome 19 using 10 kb windows. (E) Schematic of experimental plan to biochemically characterize PR-DUB in chromatin and nucleosol fractions. (F) Western blot of the indicated cell lines in either nucleosol or chromatin fractions. (G) Comparison of stoichiometry (IBAQ relative to BAP1) of PR-DUB subunits in FLAG/HA-BAP1 IP mass spectrometry purifications from nucleosol and chromatin fractions. Data are represented as mean ± SD. (H) Model of activity of PR-DUB complex at both its bound target genes and in “hit-and-run” model of highly mobile nucleosolic complex throughout the genome. See also Figure S2.

To examine H2AK119ub1 genome-wide deposition, we performed quantitative spike-in ChIP-seq analyses (Figures 2A and S2D). This revealed that both BAP1-only and RING1B-only clusters presented extensive accumulation of H2AK119ub1 that was dependent on BAP1 catalytic activity (Figure 2B). H2AK119ub1 distribution, either 3′ or 5′ to the RING1B peak areas, demonstrated a greater increase outside the peak area (Figures S2A, S2B, and S2C). This suggests that BAP1-dependent accumulation of H2AK119ub1 is caused by a spreading effect rather than a regulatory balance at Polycomb-enriched sites. Consistently, RING1B occupancy was reduced in a catalytic-dependent manner. Importantly, this occurred in the absence of *de novo* gain in PRC1 association along the genome, including at BAP1-only sites, despite a clear accumulation in H2AK119ub1 levels (Figure 2A, 2B, S2D, S2E and S2F).

We further quantified H2AK119ub1 at intergenic sites, demonstrating that it accumulated when BAP1 catalytic activity was lost (Figure 2C), a trend that is maintained at repeat elements (Figure S2G). This may suggest that BAP1 activity is devoted to preventing aberrant H2AK119ub1 accumulation genome wide. To test this at the chromosome-wide level, we divided the genome into 10 kb bins and found that H2AK119ub1 levels were significantly increased across all chromosomes (Figure 2D; chromosome 19 shown as a representative example). Importantly, while re-expression of BAP1 restored normal H2AK119ub1 levels, the catalytic-inactive C91S mutant did not. Together, this suggests that a highly mobile form of BAP1/PR-DUB maintains low levels of extragenic (extra-promoter) H2AK119ub1 and that its loss mobilizes PRC1 from its stably bound targets, contributing to spurious non-targeted H2AK119ub1 across the entire genome.

To further test this, we characterized PR-DUB in both nucleosol-associated and chromatin-associated nuclear fractions (Figure 2E). The PR-DUB subunits BAP1, FOXK2, and ASXL2 were more abundant in the nucleosol (Figure 2F). In contrast, SUZ12 and RING1B were more enriched on chromatin despite their well-established dynamic roles in modifying histones throughout the genome (Alabert et al., 2015; Ferrari et al., 2014; Fursova et al., 2019; Højfeldt et al., 2019; Huseyin and Klose, 2020; Lee et al., 2015; Youmans et al., 2018). Separation of nucleosol versus chromatin fractions with glycerol gradients revealed that BAP1 sediments in identical high-molecular-weight fractions, suggesting that the complex composition remains unaltered (Figure S2H). RING1B exists in several subcomplexes of different sizes, which indeed was detected in a broad size range. Finally, mass spectrometry (MS) analyses of FLAG-BAP1 purifications from nucleosol versus chromatin demonstrated that PR-DUB composition and stoichiometry are unaffected (Figures 2G, S2I, and S2J). Together, these biochemical data support the presence of an intact and highly mobile form of PR-DUB that can maintain low levels of H2AK119ub1 throughout the genome with “hit-and-run” activity (Figure 2H).

### PCGF3/5-PRC1 complexes are the major source of BAP1-opposed H2AK119ub1 deposition

While the knockout of RING1A/B has previously been shown to rescue BAP1-loss-mediated transcriptional changes (Campagne et al., 2019), this approach remains a poor strategy to counteract pathological deficiencies of BAP1. RING1A/B activity is generally required for cell and adult tissue viability (Chiacchiera et al., 2016; Cohen et al., 2018; Pivetti et al., 2019; Voncken et al., 2003) because it is involved in the activity of multiple PRC1 sub-complexes (Fursova et al., 2019; Scelfo et al., 2019). Therefore, we decided to delve further into the precise relationship between BAP1 and the distinct PRC1 sub-complexes in regulating H2AK119ub1 levels to uncover potential weaknesses.

**Figure 3:**
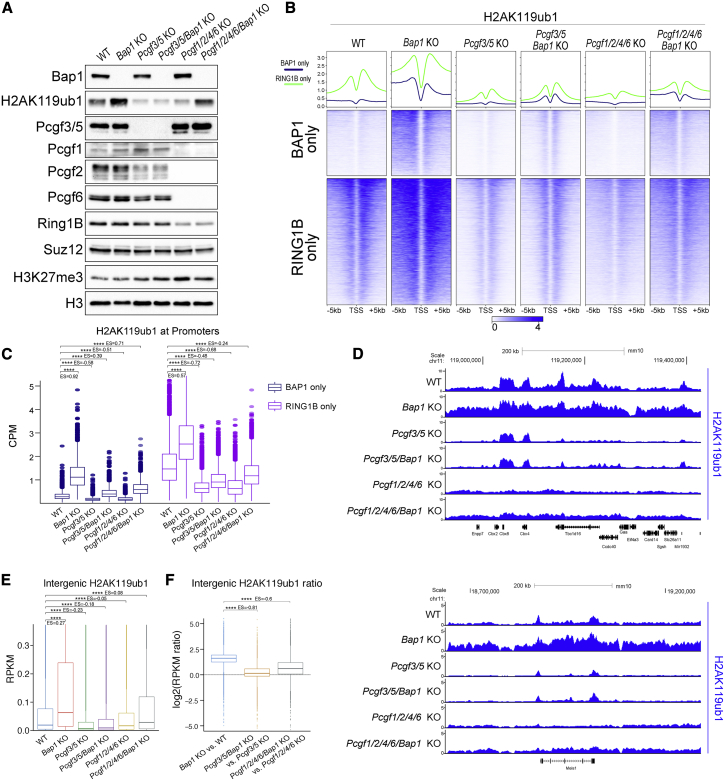
PCGF3/5-PRC1 complexes are the major source of BAP1-opposed H2AK119ub1 deposition (A) Western blot analysis with the indicated antibodies on total protein extracts from the indicated ESC lines. (B) Metaplots and heatmaps representing normalized ChIP-seq intensity for H2AK119ub1. (C) Boxplot of normalized intensity profiles for H2AK119ub1 ChIP-seq in the indicated cell lines. (D) Genome browser snapshot of H2AK119ub1 ChIP-seq in the indicated cell lines. (E) Boxplots representing H2AK119ub1 ChIP-seq RPKM levels in the indicated cell lines at intergenic sites (n = 38,068). (F) Boxplots showing the log2 fold change RPKM ratio for H2AK119ub1 in the indicated cell line comparisons at intergenic regions (n = 38,068).

Since BAP1 loss preferentially induced a genome-wide diffuse accumulation of H2AK119ub1, we reasoned that PCGF3- and PCGF5-containing complexes (PRC1.3/5) could be critical due to their roles in H2AK119ub1 diffusion and XCI (Almeida et al., 2017; Fursova et al., 2019). Using our established set of *Pcgf* knockout cell lines (Scelfo et al., 2019), we first generated a model in which the PRC1.3/5 activity was lost together with BAP1 (*Pcgf3/5* KO ± *Bap1* KO). Second, we made a model in which PRC1.3/5 was the only PRC1 activity left in ESCs (*Pcgf1/2/4/6* KO ± *Bap1* KO). Western blot analyses with these tools revealed that specific PCGF3/5 loss induced a general reduction in H2AK119ub1 levels (Figure 3A). Importantly, further loss of BAP1 was not sufficient to restore normal H2AK119ub1 levels in *Pcgf3/5* KO (Figure 3A). Only a modest increase was observed when BAP1 was depleted, suggesting that the majority but not all of BAP1-opposed H2AK119ub1 is generated by PCGF3/5 containing complexes. In comparison, *Pcgf1/2/4/6* KO cells showed milder reductions in H2AK119ub1 levels, which increased close to WT levels when BAP1 was lost (Figure 3A). Together, these results demonstrate that PCGF3/5 are primarily responsible for BAP1-opposed H2AK119ub1.

To extend these observations, we performed ChIP-seq analyses for H2AK119ub1 in these cell lines. This revealed different patterns of regulation for H2AK119ub1 deposition at RING1B target promoters (Figures 3B and 3C). H2AK119ub1 promoter levels were apparently decreased in both *Pcgf3/5* and *Pcgf1/2/4/6* KO models (in presence of BAP1). However, while loss of PRC1.3/5 affected spreading but not the normal bimodal accumulation of H2AK119ub1 at Polycomb target TSS, in the presence of PRC1.3/5 activity only (*Pcgf1/2/4/6* KO) this accumulation was lost and converted into diffuse H2AK119ub1 deposition (Figures 3B and 3D). The removal of BAP1 did not lead to any evident increase in diffuse H2AK119ub1 deposition in the absence of PCGF3/5. Consistently, when only PRC1.3/5 sub-complexes were active, BAP1 removal specifically increased the diffuse deposition of H2AK119ub1 (Figure 3D). Indeed, the quantification of H2AK119ub1 levels at all intergenic regions followed this trend (Figures 3E and 3F), demonstrating that PRC1.3/5 activity is primarily responsible for the large-scale intergenic changes in H2AK119ub1 upon *Bap1* deletion in a fashion resembling H2AK119ub1 spreading during XCI (Almeida et al., 2017; Fursova et al., 2019).

### BAP1 catalytic activity maintains stable PRC2 target association and spatial distribution of H3K27me3

**Figure 4:**
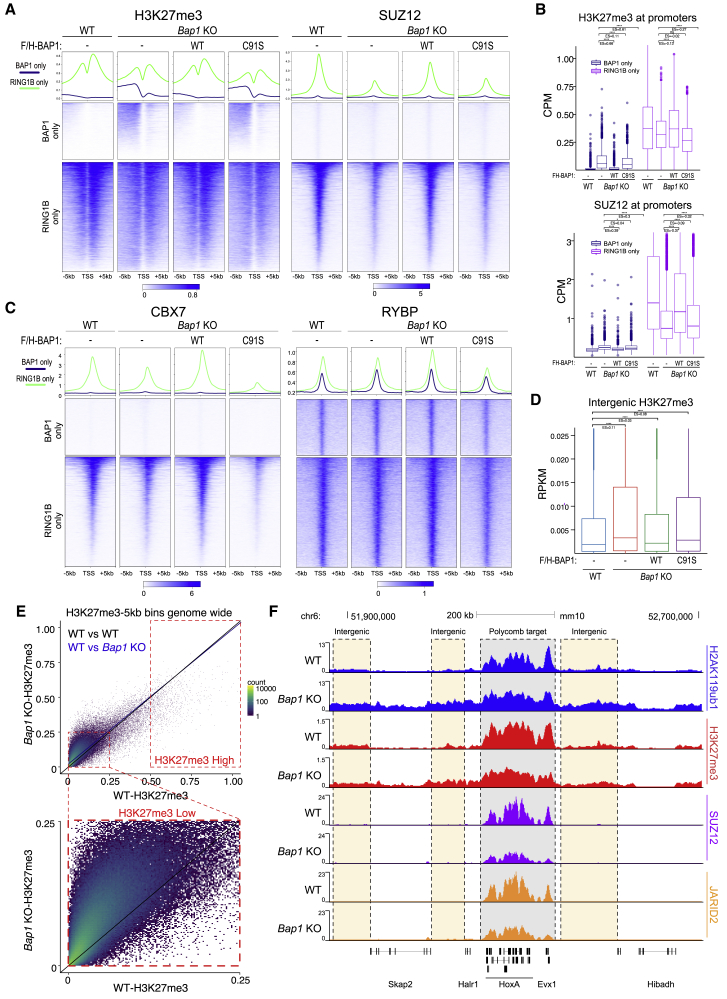
BAP1 catalytic activity maintains stable PRC2 target association and spatial distribution of H3K27me3 (A) Metaplots and heatmaps representing normalized ChIP-seq intensity for H3K27me3 (left) or SUZ12 (right). (B) Boxplot of normalized intensity profiles for H3K27me3 and SUZ12 ChIP-seq in the indicated cell lines. (C) Metaplots and heatmaps representing normalized ChIP-seq intensity for CBX7 (left) and RYBP (right) in the indicated cell lines. (D) Boxplots representing H3K27me3 ChIP-seq RPKM levels in the indicated cell lines at intergenic sites (n = 38,068). (E) Genome-wide XY scatterplot of normalized H3K27me3 ChIP-seq intensities at a resolution of 5 kb in Bap1 KO compared to WT ESCs. Each point represents one 5 kb window. (F) Genome browser snapshot of indicated ChIP-seq tracks at the HOXA locus in WT and *Bap1* KO ESCs. See also Figure S3.

There is conflicting evidence on the role played by PRC2 activity in BAP1-mediated tumor suppression (Abdel-Wahab et al., 2012; LaFave et al., 2015; Schoumacher et al., 2016). We and others have shown that H2AK119ub1 contributes to stable PRC2 binding at target genes (Blackledge et al., 2014, 2020; Healy et al., 2019; Tamburri et al., 2020) as well as for PRC2 catalytic activity (Kalb et al., 2014; Kasinath et al., 2021). ChIP-seq analyses for H3K27me3 and SUZ12 in our rescue system showed that H3K27me3 deposition followed H2AK119ub1 only to some extent. At BAP1-unique targets, loss of BAP1 or its catalytic activity caused a gain of H3K27me3 deposition 5′ to the TSS that was independent of *de novo* SUZ12 binding (Figures 4A, 4B, S3A, and S3B). The lack of 3′ invasion of H3K27me3 follows H2AK119ub1 deposition and is likely a consequence of the antagonistic relationship with RNA Polymerase activity, which maintains active transcription of these genes (Figure 1C; Beltran et al., 2016; Riising et al., 2014; Zhang et al., 2019). At RING1B target genes, we observed that both H3K27me3 and SUZ12 levels were reduced in the absence of BAP1 (Figures 4A, 4B, and S4C) despite the extensive gains in H2AK119ub1. Such displacement is dependent on BAP1 catalytic activity (Figures 4A, 4B, S3A, and S3C), in agreement with the requirement of ASXL1 for correct PRC2 activity at the HOX genes in leukemia (Abdel-Wahab et al., 2012). Levels of CBX7 were similarly reduced at the same sites (Figures 4C and S3D), consistent with its role in tethering cPRC1 by associating to H3K27me3. RYBP binding at the same sites remained unaltered, demonstrating that the more catalytically active vPRC1 forms (Blackledge et al., 2014) are maintained in the absence of BAP1 activity (Figures 4C and S3D). This supports the reduction in RING1B and the increase in H2AK119ub1 levels at target loci (Figure 2A). Thus, upon loss of BAP1 activity, PRC2 and cPRC1 are displaced from target promoters, while vPRC1 remains and sustains H2AK119ub1 deposition. Diffuse H2AK119ub1 accumulation outside of PRC1 targets (Figures S2B and S2C) outcompetes PRC2 affinity for promoters and titrates it away from its target loci. Further supporting this model, the genes that increase in expression in the absence of BAP1 tend to lose SUZ12, RING1B, and H3K27me3, while those that are downregulated maintain them (Figure S3E).

In the absence of BAP1 activity, quantifications at all intergenic sites showed that H3K27me3 underwent accumulation with a trend similar to that of H2AK119ub1 (Figure 4D). Further analysis of the entire genome confirmed this result, demonstrating that H3K27me3 accumulated exclusively at genomic regions with low levels for this PTM (Figure 4E). In contrast, the few regions that have high levels of H3K27me3 (likely Polycomb target loci) reduced this modification (Figure 4E), in agreement with PRC2 displacement. Accumulation of intergenic H3K27me3 reduced H3K36me2 intergenic levels (Figures S3F and S3G), consistent with the antagonistic relationship of PRC2 with the NSD1-3 methyltransferases (Lu et al., 2016; Streubel et al., 2018). This is reminiscent of the spurious accumulation of intergenic H3K27me3 and loss of stable Polycomb binding in H3K36M cancers (Lu et al., 2016) and may suggest a similar underlying mechanism.

Overall, intergenic gain in H2AK119ub1 titrates PRC2 away from its normal target promoters, decreasing promoter H3K27me3 concentration and increasing its indiscriminate intergenic levels (Figure 4F).

### PRC2 sub-complexes differentially contribute to the BAP1-dependent intergenic H3K27me3 deposition

**Figure 5:**
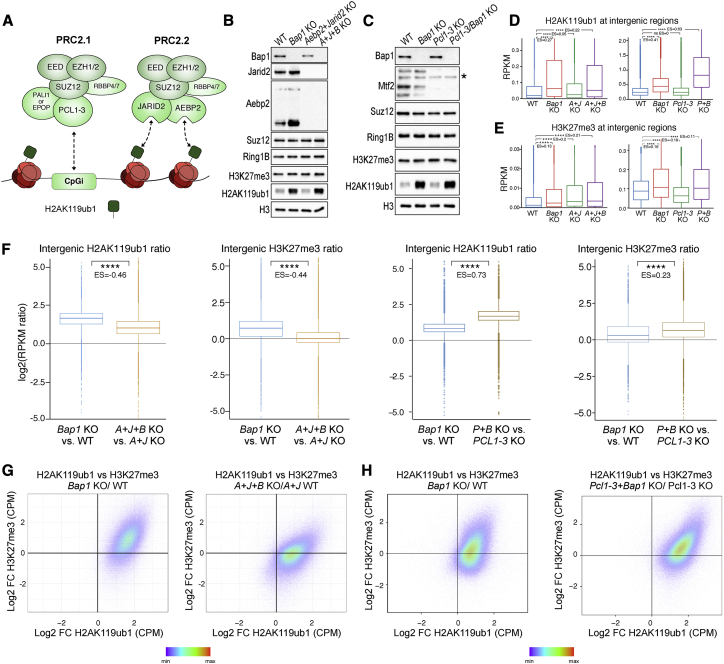
PRC2 sub-complexes differentially contribute to the BAP1-dependent intergenic H3K27me3 deposition (A) Cartoon showing divergent compositions of the PRC2.1 and PRC2.2 complexes and their differing affinities for chromatin features. (B) Western blot analysis with the indicated antibodies on total protein extracts from the *Bap1* KO, *Aebp2*+*Jarid2* (*A+J*) KO, *Aebp2*+*Jarid2*+*Bap1* (*A+J+B*) KO, and matching WT ESCs. (C) Western blot analysis with the indicated antibodies on total protein extracts from the indicated ESC lines. (D) Boxplots representing H2AK119ub1 ChIP-seq RPKM levels in the indicated cell lines at intergenic sites (n = 38,068). (E) Boxplots representing H3K27me3 ChIP-seq RPKM levels in the indicated cell lines at intergenic sites (n = 38,068). (F) Boxplots showing the log2 fold change RPKM ratio for H2AK119ub1 or H3K27me3 in the indicated cell lines (A = *Aebp2*, J = *Jarid2*, P *= Pcl1-3*) at intergenic regions (n = 38,068). (G) Genome-wide comparison of ChIP-seq signal using 5 kb windows. Log2 fold change H2AK119ub1 ChIP-seq for the *Aebp2/Jarid2/Bap1* KO versus *Aebp2/Jarid2* KO and the matching *Bap1* KO versus WT comparison (x axis) plotted against log2 fold change of H3K27me3 ChIP-seq (y axis). Each dot represents one 5 kb window. (H) Genome-wide comparison of ChIP-seq signal using 5 kb windows. Log2 fold change H2AK119ub1 ChIP-seq for the *Pcl1-3/Bap1* KO versus *Pcl1-3* KO and the matching *Bap1* KO versus WT comparison (x axis) plotted against log2 fold change of H3K27me3 ChIP-seq (y axis). Each dot represents one 5 kb window. See also Figure S4.

PRC2 sub-complexes can be broadly divided into PRC2.1 (containing PCL1-3 with either EPOP or PALI1) or PRC2.2 (containing JARID2 and AEBP2) (Beringer et al., 2016; Conway et al., 2018; Grijzenhout et al., 2016; Hauri et al., 2016). The PRC2.2 subunits JARID2 and AEBP2 have been widely reported to have an affinity for H2AK119ub1 (Cooper et al., 2016; Glancy et al., 2021; Kalb et al., 2014; Kasinath et al., 2021), whereas the PRC2.1 complex is largely targeted to chromatin through PCL1-3 affinity for CpG islands (Choi et al., 2017; Healy et al., 2019; Li et al., 2017) (Figure 5A). PRC2.2 H2AK119ub1 affinity is essential for PRC2 recruitment to the inactive X chromosome (Cooper et al., 2016), and removal of H2AK119ub1 results in a preferential loss of PRC2.2 binding compared to PRC2.1 (Blackledge et al., 2020; Tamburri et al., 2020). We knocked out *Aebp2* and *Jarid2* in the presence (*A+J*) or absence of *Bap1* (*A+J+B*; Figures 5B and S4A) and compared these with *Pcl1-3* KO ESC (Højfeldt et al., 2019) in which *Bap1* was also inactivated. As expected, H2AK119ub1 levels were increased to a similar extent in the presence or absence of AEBP2+JARID2 or PCL1-3 upon BAP1 deletion, demonstrating that the gain in PRC1 activity occurs upstream of PRC2 (Figures 5B and 5C). We performed ChIP-seq for both H2AK119ub1 and H3K27me3 and observed similar changes in *Bap1*, *Pcl1-3/Bap1* KO, and *Aebp2/Jarid2/Bap1* KO cells at RING1B target genes (Figure S4B). This showed that *Aebp2* and *Jarid2* removal did not rescue the reduction in H3K27me3 levels at Polycomb targets, while *Pcl1-3/Bap1* KO had a greater increase in H2KA119ub1 at BAP1 and RING1B targets than BAP1 KO alone. However, analysis of intergenic regions showed that loss of AEBP2 and JARID2 seems to uncouple extragenic H3K27me3 gains from H2AK119ub1 accumulation (Figures 5D–5F). While the fold change in H2AK119ub1 accumulation in non-genic regions remained similar in the presence or absence of AEBP2 and JARID2, BAP1 loss did not lead to any additive accumulation in H3K27me3 when AEBP2 and JARID2 were not expressed (Figure 5D). This opens the possibility that the remaining PRC2.1 does not efficiently sustain BAP1-opposed accumulation of H3K27me3. Please note that there is already a general gain in intergenic H3K27me3 levels in *Aebp2/Jarid2* KO cells (Figure 5E), in line with previous reports (Højfeldt et al., 2019). This is the result of a reduced tethering of core PRC2 to target promoters, which is independent of intergenic H2AK119ub1 accumulation. Importantly, H3K27me3 accumulated at extragenic sites in both the presence (*Bap1* KO) or absence (*Pcl1-3/Bap1* KO) of PCL1-3 expression (Figures 5D–5F), demonstrating that the remaining PRC2 activity is capable of sustaining BAP1-dependent H3K27me3 extragenic accumulation (Figure S4C). Indeed, *Pcl1-3/Bap1* KO displayed an even greater H3K27me3 accumulation that is likely dependent on an established switch in PRC2.2 stoichiometry (Healy et al., 2019), promoting PRC2 affinity for H2AK119ub1.

The uncoupling of intergenic H3K27me3 gain from H2AK119ub1 spreading can be better visualized through scatterplots of the entire genome, where fold changes in H2AK119ub1 were plotted against H3K27me3 (Figures 5G and 5H). Importantly, this analysis demonstrated a high concordance in the genomic regions that gained both H2AK119ub1 and H3K27me3 (Figures 5G and 5H). When the same analysis was performed in the absence of AEBP2/JARID2, H2AK119ub1 accumulation was uncoupled from H3K27me3 (Figure 5G), whereas *Pcl1-3* KO largely resembled the matching *Bap1 KO* plot (Figure 5H). This supports the possibility that distinct PRC2 sub-complexes contribute differently to BAP1-dependent H3K27me3 spreading, with a prominent role for the PRC2.2 form containing JARID2 and AEBP2.

### Diffuse H2AK119ub1 accumulation causes global chromatin compaction

**Figure 6:**
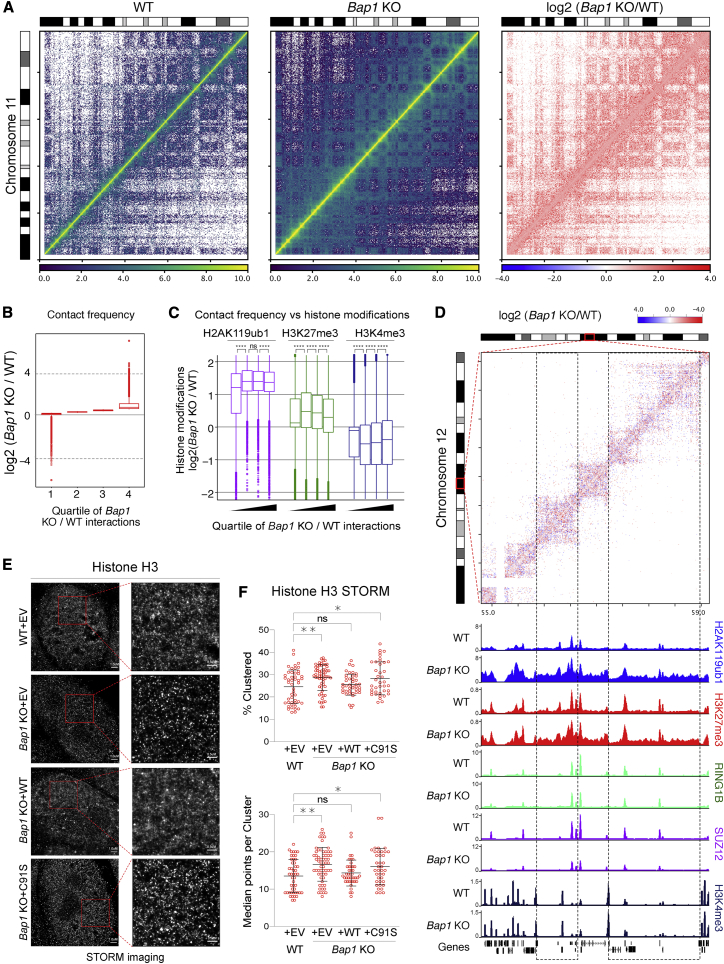
Diffuse H2AK119ub1 accumulation causes global chromatin compaction (A) Ice-normalized HiC contact matrix of the entire chromosome 11 in WT, *Bap1* KO, and log2 fold change (BAP1 KO/WT) at 250 kbp resolution using two pooled HiC replicates. (B) Boxplot of contact frequency of log2 fold change (*Bap1* KO/WT) ratios divided into quartiles using two pooled HiC replicates. (C) Boxplot of the log2 fold change (*Bap1* KO/WT) ratio of the indicated histone modifications within the quartiles defined in (B). Wilcoxon test was used to ascertain significance. (D) Top: Log2 fold change (*Bap1* KO/WT) HiC contact matrix of the indicated region of chromosome 12 (54.8–59.15 Mb) at 10 kb resolution. Bottom: Genome browser snapshot of indicated ChIP-seq tracks at the same region of chromosome 12. (E) Representative stochastic optical reconstruction microscopy (STORM) images of the indicated cell lines stained with histone 3. Scale bars of 1 μm (left column) and 0.5 μm (right column) are shown. (F) STORM quantifications of percentage clustered (top) and median points per cluster (bottom) of H3. Data are represented as mean ± SD. See also Figure S5.

Since the activity of the PRC1.3/5 complexes has been linked to large-scale chromatin condensation through XCI (Almeida et al., 2017), we wondered whether diffuse accumulation of H2AK119ub1 coupled to H3K27me3 deposition could lead to changes in 3D chromatin organization and compaction. For this, we performed *in situ* HiC analysis in WT and *Bap1* KO ESCs and found that loss of BAP1 induces a general gain in contacts across the entire genome, as exhibited in the representative contact matrices for chromosome 11 (Figures 6A and S5A). Splitting these contact changes into quartiles based on the fold change, we found that all four quartiles have an average enrichment in *Bap1* KO (Figures 6B and S5B). This showed that over three-quarters of the genome acquired a more compact configuration in the absence of BAP1, without major changes in contact size distribution or inter/intra-chromosome contact frequency (Figure S5C). Correlation of changes in histone modifications within these quartiles showed that the largest gain in H2AK119ub1 and H3K27me3 was exhibited at the mid-high quartiles, coinciding with the largest loss in H3K4me3 (Figure 6C). This hints at a model where the broad gains in Polycomb modifications coincide with a reduction in active histone modifications. Overlaying HiC matrices with ChIP-seq profiles highlighted the spreading of H2AK119ub1 and H3K27me3 relative to compaction and some local reductions in H3K4me3 deposition (Figure 6D). Importantly, loss of BAP1 affected only the general compaction state of chromatin without altering the status and boundaries of topology-associated domains (TADs). Quantitative MS analysis of chromatin-associated proteins revealed an enrichment for repressive and heterochromatic factors upon BAP1 loss, coupled with a displacement of factors associated with active transcription and replication (Figure S5D). This supports the possibility that loss of BAP1 creates a more compact chromatin environment.

*In vivo* nucleosomes are organized in clusters (clutches) of different sizes interspersed by nucleosome-free DNA (Ricci et al., 2015). To investigate if the observed compaction affected nucleosome organization, we performed histone H3 staining followed by stochastic optical reconstruction microscopy (STORM) imaging to identify and quantify nucleosome clutches in our rescue system. Representative images of STORM reconstruction highlighted that the chromatin of *Bap1* KO ESCs displayed brighter and less diffuse nucleosome clusters (Figure 6E). This effect was rescued by BAP1 re-expression and was dependent on its catalytic activity (Figure 6E). Quantification of histone H3 molecule (point) localization and grouping into clusters (Figure S5E) further demonstrated that histone H3 becomes more densely packed when BAP1 activity is lost (Figure 6F). Additionally, we observed a reduction in nuclear area in the absence of BAP1 or its catalytic activity (Figure S5F). Together, these results demonstrate that BAP1 preserves low levels of H2AK119ub1 across the genome to counteract a general compaction of chromatin.

### Bap1 null mesothelioma growth requires PCGF3/5-dependent H2AK119ub1 accumulation

**Figure 7:**
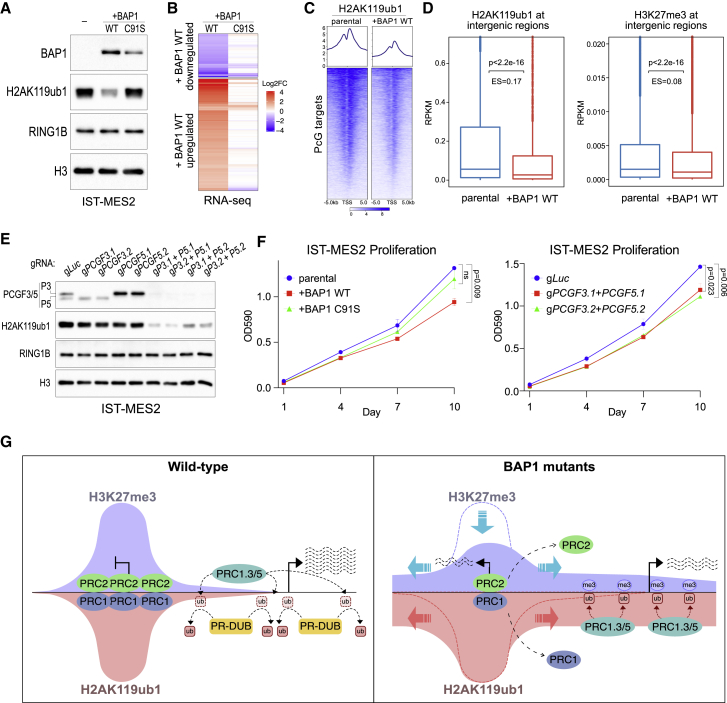
Bap1 null mesothelioma growth requires PCGF3/5-dependent H2AK119ub1 accumulation (A) Western blot using the indicated antibodies in whole-cell lysates of the BAP1 null IST-MES2 mesothelioma cell line with either parental or BAP1 WT/C91S overexpression. (B) RNA-seq heatmap of those genes differentially expressed in +BAP1 WT IST-MES2 versus parental IST-MES2. (C) Metaplots and heatmaps representing normalized ChIP-seq intensity for H2AK119ub1 in the indicated cell lines. (D) Boxplots representing H2AK119ub1 (left) or H3K27me3 (right) ChIP-seq RPKM levels in the indicated cell lines at intergenic sites. (E) Western blot using the indicated antibodies in IST-MES2 cell line whole-cell lysates following knockout of *PCGF3* or *PCGF5* in the indicated combinations. (F) Growth curves measured using crystal violet staining (λ = 590 nm) of the indicated cell lines. Data are represented as mean ± SD. (G) Model of the dual role of BAP1 mode of action on transcription. BAP1 is essential for the spatial maintenance of H2AK119ub1 and H3K27me3. Spurious redistribution of these in the absence of BAP1, directed by the PCGF3/5-PRC1 and PRC2.2 complexes, promotes chromatin compaction and a general repression of transcription (Trithorax phenotype) while simultaneously allowing derepression of selected Polycomb target genes (Polycomb phenotype).

To investigate the pathophysiological conservation and relevance of this model, we made use of a BAP1 null mesothelioma cell line (IST-MES2) in which we stably re-expressed BAP1 WT or the catalytic C91S mutant. Western blot confirmed that restoring BAP1 activity depletes H2AK119ub1 levels in a catalytic-dependent manner (Figure 7A). RNA-seq analysis showed that DEGs induced by BAP1 in IST-MES2 cells were dependent on BAP1’s catalytic activity (Figure 7B). ChIP-seq for H2AK119ub1 confirmed that restoring BAP1 activity reduced H2AK119ub1 levels at both Polycomb target promoters (Figure 7C) and intergenic sites (Figure 7D). Importantly, BAP1 restoration also reduced H3K27me3 levels at extragenic sites (Figure 7D), generalizing the model to pathologically relevant conditions. Consistently, combined knockout of *PCGF3/5* extensively reduced global H2AK119ub1 levels. While individual PCGF3 or PCGF5 loss has only a minor effect, highlighting redundancies, their double knockout profoundly affected H2AK119ub1 in this system (Figure 7E). Importantly, BAP1 restoration in IST-MES2 cells significantly slowed cell growth in a catalytic-dependent manner, demonstrating that mesothelioma tumors remain addicted to the loss of BAP1 activity (Figure 7E). In line with this, loss of PCGF3/5 phenocopied the growth-inhibitory effect of BAP1 restoration (Figure 7E). This validates our model and further suggests that this mechanism could be an attractive therapeutic strategy to restore H2AK119ub1 levels to a non-pathological state in BAP1 null contexts.

We propose a model (Figure 7G) in which the primary role of BAP1 is in removing non-specific PCGF3/5-dependent H2AK119ub1 throughout the genome. The absence of BAP1 leads to accumulation of H2AK119ub1 intergenically, which titrates PRC2 away from its target genes. This depletion of Polycomb complexes from their target sites allows aberrant activation of transcription, while simultaneously causing repressive chromatin compaction elsewhere. This model is further supported by key observations reported in a recent study from the Klose group (Fursova et al., 2021). This indirect regulation of transcription and chromatin conformation may help explain the tumor-suppressive functions of BAP1.

## Discussion

These results uncover a BAP1 mode of action that can be highly relevant to disease, linking loss-of-function mutations to disruption of transcriptional programs in tumors. Activation of a number of Polycomb target genes may mimic aspects of PRC2 loss-of-function mutations and events, which are a common feature of many cancers (Conway et al., 2015; Jain et al., 2019; Lee et al., 2014; Ntziachristos et al., 2012; Piunti et al., 2017). Loss of Polycomb silencing is also observed in some ASXL1 mutant myeloid leukemias, further supporting this model (Abdel-Wahab et al., 2012). Titration of Polycombs from their canonical sites is an emerging theme, as it has been shown that deletion of BAF complex subunits paradoxically reduces Polycomb occupancy and silencing from their target sites (Weber et al., 2021). Like PR-DUB, BAF complex subunits are also frequently mutated in several cancers (Bracken et al., 2019), suggesting recurrent alternate mechanisms, outside of direct PRC2 mutations, for loss of Polycomb transcriptional control.

The role of BAP1 in preventing intergenic spreading of H3K27me3 and chromatin compaction is reminiscent of EZH2 gain-of-function cancers such as non- Hodgkin’s lymphoma (Morin et al., 2011) in which genome-wide H3K27me3 accumulation correlates with local increases in compaction and transcriptional inactivation of affected TADs (Donaldson-Collier et al., 2019). This paradigm of intergenic spreading of H3K27me3 is found in several other cancer types, such as chondroblastomas featuring H3K36M mutations causing diffuse accumulation of H3K27me3, loss of H3K36me2, and titration of Polycomb binding away from promoters (Lu et al., 2016). A similar phenotype is observed in H3.3G34 mutant osteosarcomas, in which intergenic H3K27me3 accumulates to repress distal regulatory elements (Jain et al., 2020).

The specific contribution of either maintenance of Polycomb repression or inhibition of chromatin compaction in BAP1’s tumor-suppressive role remains an open issue to be uncoupled. Nonetheless, our genetic analyses for H2AK119ub1 and H3K27me3 regulation suggest potential strategies for synthetic lethality relevant in a cancer context. This may not be limited to cancer, as neurodevelopmental disorders featuring mutations in the ASXL subunits of PR-DUB may also be affected through the same mechanisms (Bainbridge et al., 2013; Hoischen et al., 2011). Intriguingly, one of the few PRC1 subunits mutated in neurological and intellectual disability disorders is AUTS2, which is part of the PCGF3/5 complexes (Gao et al., 2014; Kalscheuer et al., 2007; Si et al., 2016). This may suggest that a careful balance in the levels of intergenic or broad domains of H2AK119ub1 is essential to maintain transcriptional homeostasis during neurodevelopment.

While it is clear that Polycomb-mediated histone modifications are involved in transcriptional repression (Blackledge et al., 2020; Pengelly et al., 2013; Tamburri et al., 2020), how exactly H2AK119ub1 participates in this remains unclear. Although H2AK119ub1 may promote PRC2.2 recruitment, PRC2 loss in ESCs has little effect on transcription in contrast to H2AK119ub1 loss (Blackledge et al., 2020; Lavarone et al., 2019; Tamburri et al., 2020). Thus, there is some underappreciated role of H2AK119ub1 in blocking transcription, perhaps through its antagonistic relationship with RNA Pol II initiation (Dobrinić et al., 2020). It is possible that the silencing role of H2AK119ub1 causes widespread repressive pathogenic effects in BAP1 mutant cancers. The contribution of PRC1 in regulating the 3D epigenome has been investigated primarily through non-catalytic roles of canonical PRC1 and the compaction-related functions of the PHC and CBX subunits (Isono et al., 2013; Kundu et al., 2017; Plys et al., 2019; Tatavosian et al., 2019). Whether H2AK119ub1 plays a role in this remains unclear due to the use of hypomorphic RING1B mutants (Boyle et al., 2020; Kundu et al., 2017). The essential role of PCGF3/5 in X chromosome inactivation and the requirement of JARID2 for XCI both suggest that H2AK119ub1 is involved in this type of compaction (Almeida et al., 2017; Cooper et al., 2016). However, whether that role extends to somatic chromosomes and local 3D structures such as TADs and Polycomb bodies is not yet understood. Our data suggest that diffuse intergenic levels of H2K119ub1 can stimulate local compaction of chromatin in a repressive fashion, similarly to XCI.

Finally, the dual function that we described for BAP1 in both activation and repression of Polycomb target genes helps resolve a puzzling issue in developmental biology. The Polycomb and Trithorax *in vivo* phenotypes in Calypso and ASXL mutants in *Drosophila* and mice (Baskind et al., 2009; Fisher et al., 2010; Scheuermann et al., 2010) have been confounding mechanistically until now. It is clear that in the context of development, a fine balance between H2AK119ub1 levels is essential to maintain normal gene expression programs.

### Limitations of the study

While our results suggest that JARID2 and AEBP2 play an important role in decommissioning PRC2 activity from promoters, triggering diffused H3K27me3 accumulation upon BAP1 loss, it is possible that core PRC2 forms (EZH1/2, SUZ12, EED, and RBBP4/7) or other H2AK119ub1-dependent crosstalk mechanisms may also contribute to this process. For instance, H3K36me2 catalysis by NSD1-3 enzymes is efficient only when H2AK119 is unmodified (Li et al., 2021). Therefore, it could be possible that a H2AK119ub1-dependent H3K36me2 reduction contributes to increase H3K27me3 levels, enhancing PRC2 catalysis. However, this conflicts with the accumulation of H3K27me3 levels reported in NSD1-depleted cells at Polycomb target promoters (Streubel et al., 2018), which is in contrast with the reduction of H3K27me3 levels found at the same sites when BAP1 activity is lost. Together, these observations primarily favor a titration model that is further supported by reduced PRC2 occupancy at target sites. Alternatively, a number of new H2AK119ub1 sensors have been reported recently (McBride et al., 2020; Weinberg et al., 2021), but their effective role in PR-DUB- and PRC1-related processes remains to be fully established.

## STAR★Methods

### Key resources table

**Table:** 

REAGENT or RESOURCE	SOURCE	IDENTIFIER
**Antibodies**		

Rabbit monoclonal anti-BAP1 (D7W70)	Cell Signaling	Cat #13271; RRID: AB_2798169
Rabbit monoclonal anti-SUZ12 (D39F6)	Cell Signaling	Cat #3737; RRID: AB_2196850
Rabbit monoclonal anti-H3K27me3 (C36B11)	Cell Signaling	Cat #9733; RRID: AB_2616029
Rabbit monoclonal anti-H2AK119ub1 (D27C4)	Cell Signaling	Cat #8240; RRID: AB_10891618
Rabbit monoclonal anti-JARID2 (D6M9X)	Cell Signaling	Cat #13594; RRID: AB_2798269
Rabbit monoclonal anti-AEBP2 (D7C6X)	Cell Signaling	Cat #14129; RRID: AB_2798398
Rabbit monoclonal anti-HA (C29F4)	Cell Signaling	Cat #3724; RRID: AB_10693385
Rabbit monoclonal anti-ASXL1 (D1B6V)	Cell Signaling	Cat #52519; RRID AB_2799415
Rabbit monoclonal anti-KDM1B/LSD2 (E1R60)	Cell Signaling	Cat #54576; RRID: AB_2799465
Rabbit polyclonal anti-CBX7	Abcam	Cat #ab21873; RRID: AB_726005
Rabbit monoclonal anti-PCGF3/5	Abcam	Cat #ab201510
Rabbit polyclonal anti-H3	Abcam	Cat #ab1791; RRID: AB_302613
Rabbit polyclonal anti-H3K36me2	Abcam	Cat #ab9049; RRID: AB_1280939
Rabbit polyclonal anti-H3K27ac	Abcam	Cat #ab4729; RRID: AB_2118291
Rabbit monoclonal anti-RING1B-pS168	Abcam	Cat #ab234421
Rabbit polyclonal anti-ASXL2	Bethyl Laboratories	Cat #A302-037A; RRID: AB_1576481
Rabbit polyclonal anti-HCFC1	Bethyl Laboratories	Cat #A301-400A; RRID: AB_961015
Rabbit polyclonal anti-FOXK2	Bethyl Laboratories	Cat #A301-730A; RRID: AB_1211449
Rabbit polyclonal anti-MTF2	Proteintech	Cat # 16208-1-AP; RRID: AB_2147370
Mouse monoclonal anti-FLAG	Merck (Sigma-Aldrich)	Cat # F3165; RRID: AB_259529
Rabbit polyclonal anti-RYBP (DEDAF)	Merck (Sigma-Aldrich)	Cat #PRS2227; RRID: AB_1847589
Rabbit polyclonal anti-H2A	Merck (Sigma-Aldrich)	Cat #07-146; RRID: AB_310394
Rabbit polyclonal anti-MBD6	Merck (Sigma-Aldrich)	Cat # SAB1305225-40TST
Rabbit polyclonal anti-OGT	Santa-Cruz	Cat #sc-32921; RRID: AB_2156938
Rabbit polyclonal antI-PCGF1	Scelfo et al., 2019	N/A
Rabbit polyclonal anti-PCGF2	Scelfo et al., 2019	N/A
Rabbit polyclonal anti-PCGF6	Scelfo et al., 2019	N/A
Rabbit polyclonal anti-RING1B	Chiacchiera et al., 2016	N/A
Rabbit polyclonal anti-UTX	Agger et al., 2007	N/A
Anti-FLAG M2 affinity gel	Merck (Sigma-Aldrich)	Cat #A2220; RRID: AB_10063035
Rabbit IgG control antibody	Merck (Sigma-Aldrich)	Cat #I5006; RRID: AB_1163659

**Bacterial and virus strains**		

One Shot TOP10 Chemically Competent *E. coli*	Thermo Fisher Scientific (Invitrogen)	Cat #404010
One Shot Stbl3 Chemically Competent *E. coli*	Thermo Fisher Scientific (Invitrogen)	Cat #737303

**Chemicals, peptides, and recombinant proteins**		

Leukemia Inhibitory Factor	Pasini Laboratory	N/A
CHIR-99021	Aurogen	Cat #S1263
PD-0325901	Aurogen	Cat #S1036
Lipofectamine 2000 Transfection Reagent	Thermo Fisher Scientific (Invitrogen)	Cat #11668027
3x FLAG Peptide	Merck (Millipore)	Cat #F4799
Ethylene glycol-bis(succinic acid N-hydroxysuccinimide ester)	Merck (Sigma-Aldrich)	Cat #E3257
IGEPAL CA-630	Merck (Sigma-Aldrich)	Cat #18896
DpnII	NEB	Cat #R0453
DNA Polymerase I, Large Klenow Fragment	NEB	Cat #M0210
Biotin-dATP	Thermo Fisher Scientific (Invitrogen)	Cat #19524016
Streptavidin C-1 Beads	Thermo Fisher Scientific (Invitrogen)	Cat #65001
TN5	Pasini Laboratory	N/A

**Critical commercial assays**		

Agilent High Sensitivity DNA kit	Agilent	Cat #5067-4626
QIAquick PCR purification kit	QIAGEN	Cat #28104
Quick-RNA MiniPrep extraction kit	Zymo research	Cat #R1055

**Deposited data**		

Raw Next-gen sequencing files	This paper	GEO: GSE162739
Deposited raw imaging data	This paper	Mendeley Data: https://doi.org/10.17632/yv6c2s8jtc.1

**Experimental models: Cell lines**		

Mouse: ES cell line E14	Pasini Laboratory	N/A. Strain of origin 129P2/Ola
Mouse: BAP1 KO c1 ES cell line	This paper	N/A. Strain of origin 129P2/Ola
Mouse: BAP1 KO c2 ES cell line	This paper	N/A. Strain of origin 129P2/Ola
Mouse: BAP1 KO+EV (pCAG-empty vector) ES cell line	This paper	N/A. Strain of origin 129P2/Ola
Mouse: BAP1 KO+BAP1 WT (pCAG-BAP1 WT) ES cell line	This paper	N/A. Strain of origin 129P2/Ola
Mouse: BAP1 KO+BAP1 C91S (pCAG-BAP1 WT) ES cell line	This paper	N/A. Strain of origin 129P2/Ola
Mouse: E14+EV (pCAG-empty vector) ES cell line	This paper	N/A. Strain of origin 129P2/Ola
Mouse: E14+BAP1 WT (pCAG-BAP1 WT) ES cell line	This paper	N/A. Strain of origin 129P2/Ola
Mouse: PCGF3/5 KO ES cell line	Scelfo et al., 2019	N/A. Strain of origin 129P2/Ola
Mouse: PCGF3/5 /BAP1 KO ES cell line	This paper	
Mouse: PCGF1/2/4/6 KO ES cell line	Scelfo et al., 2019	N/A. Strain of origin 129P2/Ola
Mouse: PCGF1/2/4/6/ BAP1 KO ES cell line	This paper	N/A. Strain of origin 129P2/Ola
Mouse: AEBP2/JARID2 KO ES cell line	This paper	N/A. Strain of origin 129P2/Ola
Mouse: AEBP2/JARID2/BAP1 KO ES cell line	This paper	N/A. Strain of origin 129P2/Ola
Mouse: TCF2.2 ES cell line	Højfeldt et al., 2019	N/A
Mouse: TCF2.2 ES cell line BAP1 KO	This paper	N/A
Mouse: TCF2.2 ES cell line PCL1-3 KO	Højfeldt et al., 2019	N/A
Mouse: TCF2.2 ES cell line PCL1-3 /BAP1 KO	This paper	N/A
Human: 293T	Pasini Laboratory	N/A
Human: Phoenix-Ampho	Nolan Laboratory	N/A
Human: IST-MES2 cell line	ICLC	HTL01007
Drosophila: S2 cell line	ATCC	ATCC CRL-1963

**Oligonucleotides**		

See Table S3.	This paper	N/A

**Recombinant DNA**		

pSpCas9(BB)-2A-GFP (PX458)	Zhang Laboratory	Addgene plasmid #48138
pCAG-2XFLAG-HA	Pasini Laboratory	N/A
pLENTI-Cas9	Vakoc Laboratory	Addgene plasmid #52962
LRG2.1 Puro	Zhang Laboratory	Addgene plasmid #125594
LRG2.1 Neo	Zhang Laboratory	Addgene plasmid #125593
pCAG-2XFLAG-HA-BAP1 WT	This paper	N/A
pCAG-2XFLAG-HA-BAP1 C91S	This paper	N/A
pMINKIO FLAG/HA-BAP1 WT	This paper	N/A
pMINKIO FLAG/HA-BAP1 C91S	This paper	N/A
pMINKIO empty vector	Bracken Laboratory	N/A

**Software and algorithms**		

Bowtie v1.2.2	Langmead et al., 2009	http://bowtie-bio.sourceforge.net/index.shtml
MACS2 v2.1.1	Zhang et al., 2008	https://github.com/macs3-project/MACS
ChIPpeakAnno v3.15	Zhu et al., 2010	N/A
VennDiagram v1.6.20	Chen and Boutros, 2011	https://www.rdocumentation.org/packages/VennDiagram
DeepTools 3.1	Ramírez et al., 2016	https://deeptools.readthedocs.io/en/latest/
RepeatMasker-open 4.0	https://www.repeatmasker.org/	N/A
STAR v2.7	N/A	N/A
DESeq2 v1.24	Love et al., 2014	https://bioconductor.org/packages/release/bioc/html/DESeq2.html
MaxQuant software (version 1.6.2.3)	Tyanova et al., 2016a	https://maxquant.org/
Perseus v1.6.2.3	Tyanova et al., 2016b	N/A
Graphpad PRISM v8.4.3	N/A	https://www.graphpad.com/scientific-software/prism/
HiCpro	Servant et al., 2015	https://github.com/nservant/HiC-Pro
HiCpro2juicebox	Robinson et al., 2018	N/A
HiCplotter	Akdemir and Chin, 2015	N/A
CAML	Williamson et al., 2020	https://gitlab.com/quokka79/caml
ThunderSTORM	Ovesný et al., 2014	N/A

### Resource availability

#### Lead Contact

Further information and requests for resources and reagents should be directed to and will be fulfilled by the lead contact, Diego Pasini (diego.pasini@ieo.it).

#### Materials Availability

ESC lines generated in this study are available from the lead contact upon request.

#### Data and code availability

• Sequencing data have been deposited on the GEO repository with code GEO: GSE162739 and will be publicly available as of the date of publication. Accession numbers are listed in the key resources table.
• Original images have been deposited to Mendeley data and are available at Mendeley Data: https://doi.org/10.17632/yv6c2s8jtc.1.
• This paper does not report original code.
• Any additional information required to re-analyze the data reported in this paper is available from the lead contact upon request.

### Experimental model and subject details

#### Cell lines and cell culture

mESCs (both E14 and TCF background) were grown on 0.1% gelatin-coated dishes in 2i/LIF-containing GMEM medium (Euroclone) supplemented with 20% fetal calf serum (Euroclone), 2 mM glutamine (GIBCO), 100 U/mL penicillin, 0.1 mg/mL streptomycin (GIBCO), 0.1 mM non-essential amino acids (GIBCO), 1 mM sodium pyruvate (GIBCO), 50 μM β-mercaptoethanol phosphate buffered saline (PBS; GIBCO), 1000 U/mL leukemia inhibitory factor (LIF; produced in-house), and GSK3β and MEK 1/2 inhibitors (ABCR GmbH) to a final concentration of 3 μM and 1 μM, respectively. For ATRA stimulation of ESCs, cells were washed twice with PBS 24 h after seeding and media was replaced with ESC media lacking LIF, GSK3β and MEK1/2 inhibitors, and instead supplemented with 1 μM ATRA for 24 h.

To generate stable KO cell lines, 10ug pX458 2.0 plasmid pairs (Addgene) encoding Cas9 and sgRNAs (Table S3) were transfected using Lipofectamine 2000 (Invitrogen), according to manufacturer’s instruction. Sorting of GFP positive cells was carried out 48 h after transfection and 1000 cells were seeded onto a 15-cm dish. Clones were isolated 10-14 days later and grown further before screening for knockout by western blot. For rescue clone generation, mESCs were transfected with 10ug pCAG vectors encoding 2xFlag-HA-tagged BAP1 wild-type or BAP1 C91S using Lipofectamine 2000 (ThermoFisher Scientific), according to manufacturer’s instructions. 24 h post-transfection puromycin selection (1μg/mL) was added for a further 24 h. Cells were then split to clonal density (∼1:40) onto a 15cm plate. Clones were isolated 10-14 days later and grown further before screening for rescue allele expression by western blot.

IST-MES2 were cultured with DMEM media (Euroclone) supplemented with 10% fetal bovine serum (Euroclone), 2 mM glutamine (GIBCO), 100 U/mL penicillin, 0.1 mg/mL streptomycin (GIBCO), 0.1 mM non-essential amino acids (GIBCO), Lentiviral particles for LRG2.1 Puro or Neo (addgene #125594 and #125593, a gift from Christopher Vakoc (Tarumoto et al., 2020))), or pLENTI-Cas9 (addgene #52962, a gift from Feng Zhang (Sanjana et al., 2014)) were generated by calcium phosphate transfection of 8 μg these plasmids into HEK293T with 6 μg psPAX2 and 4 μg pMD2.G. Media was changed 24 h post-transfection and subsequently viral particle containing media was collected after 48 h before 0.45 μm filtering and storage at −80°C. IST-MES2 cells were lentivirally infected by seeding 500,000 cells to 6 well plates for 1 h before spin infection with virus, in the presence of Polybrene, at room temperature, at 1700RPM for 30 min. 24 h after infection cells were split into selection with Puromycin (1 μg/mL), G418 (0.5mg/mL) or Blasticidin (10 μg/mL). IST-MES2 Cas9 cells were first generated using pLENTI-Cas9 virus, and validated for Cas9 expression by western blot, before infection with LRG2.1 virus. gRNA targeting firefly Luciferase gene was used as a negative control. For retroviral infection Phoenix amphoteric cells were transfected by calcium phosphate transfection with pMINKIO expression vector containing with BAP1 WT or C91S mutant ORF. 48 and 72 h after transfection supernatant from these transfections was filtered at 0.45 μm before addition, with Polybrene, to the target IST-MES2 cells. 24 h after final infection cells were split into selection with Puromycin (1 μg/mL).

Crystal violet growth curves were performed by seeding, in triplicate, 50,000 cells to 12 well plates for IST-MES2 cell lines. Plates were stained on days 1, 4, 7 and 10. To stain cells, wells were washed once with PBS before addition of Crystal violet stain (0.5% crystal violet, 35% ethanol) for ten minutes to stain and fix cells. Wells were washed x4 with PBS before allowing to dry overnight. To quantify staining 10% acetic acid was added to each well and plates were placed on a rocker for 20 min to solubilize dye. The sample was subsequently diluted 1:5 with water before quantification at O.D 590 on a spectrophotometer. Significance was measured at D10 using unpaired Welch’s t test.

### Method details

#### Western Blot

For western blot analysis on total protein lysates, mESCs were lysed and sonicated in ice-cold S300 buffer (20 mM Tris-HCl pH 8.0, 300 mM NaCl, 10% glycerol, 0.2% NP40) and supplemented with protease inhibitors (Roche). Precipitates were removed by centrifugation. Lysates were then resuspended in Laemmli sample buffer and boiled for 5 min. Protein lysates were separated on SDS-PAGE gels and transferred to nitrocellulose membranes. After probing with the suitable primary and secondary antibodies, chemiluminescence signals were captured with the ChemiDoc Imaging System (Bio-Rad).

#### Antibodies

Western blot and ChIP analyses were performed with: anti-BAP1 (D7W70; Cell Signaling), anti-Suz12 (D39F6; Cell Signaling Technology), anti-FLAG (F3165; Sigma-Aldrich), anti-H3K27me3 (9733; Cell Signaling Technology), anti-H2AK119ub1 (8240; Cell Signaling Technology), anti-H3 (ab1791; Abcam), anti-H2A (07-146; Sigma-Aldrich), anti-RING1B (homemade (Chiacchiera et al., 2016)), anti-PCGF1, anti-PCGF2, and anti-PCGF6 (Pasini’s lab, homemade (Scelfo et al., 2019)), anti-JARID2 (13594, Cell Signaling Technology), anti-AEBP2 (14129; Cell signaling Technology), anti-HA (C29F4, Cell Signaling Technology), anti-ASXL2 (A302-037A, Bethyl), anti-ASXL1 (D1B6V, Cell Signaling Technology) anti-KDM1B/LSD2 (E1R60, Cell Signaling Technology), anti-OGT (SC-32921, Santa-Cruz), anti-HCFC1 (A301-400A, Bethyl), anti-FOXK2 (A301-730A, Bethyl), anti-MBD6 (SAB1305225-40TST, Sigma-Aldrich), anti-H3K27ac (AB4729, Abcam) anti-PCGF3/5 (AB201510, Abcam), anti-UTX (Helin lab, homemade (Agger et al., 2007)), anti-MTF2 (Proteintech 16208-1-AP), anti-CBX7 (abcam, ab21873), anti-RYBP/DEDAF (Sigma-Aldrich, PRS2227), anti-H3K36me2 (abcam, ab9049), anti-RING1B-pS168 (abcam, ab234421).

#### Chromatin immunoprecipitation (ChIP)

ChIP experiments were performed according to standard protocols as described previously (Ferrari et al., 2014). For all ChIPs, except HA, 1% formaldehyde cross-linked chromatin was sheared to 500–1000 bp fragments by sonication. For HA ChIPs cells were fixed in 2.5mM EGS for 50 min before the addition of formaldehyde to 1% for a further 10 min, before proceeding to sonication. Chromatin was then incubated overnight in IP buffer (33 mM Tris-HCl pH 8, 100 mM NaCl, 5 mM EDTA, 0.2% NaN_3_, 0.33% SDS, 1.66% Triton X-100) at 4°C with the indicated antibodies (5 μg antibodies/ 500 μg chromatin). For histone modifications ChIPs, 250 μg of chromatin supplemented with 5% spike-in of S2 *Drosophila* chromatin (prepared in the same manner) and 3 μg of antibodies was used. The next day, chromatin lysates were incubated for 3 h with protein-G Sepharose beads (GE Healthcare). Beads were washed 3 × with low-salt buffer (150 mM NaCl, 20 mM Tris-HCl pH 8, 2 mM EDTA, 0.1% SDS, 1% Triton X-100) and 1 × with high-salt buffer (500 mM NaCl, 20 mM Tris-HCl pH 8, 2 mM EDTA, 0.1% SDS, 1% Triton X-100), and then re-suspended in de-crosslinking solution (0.1 M NaHCO3, 1% SDS). DNA was purified with QIAquick PCR purification kit (QIAGEN) according to manufacturer’s instructions. DNA libraries were prepared with 2–10 ng of DNA using an in-house protocol (Blecher-Gonen et al., 2013) by the IEO genomic facility and sequenced on an Illumina HiSeq 2000. ChIP-seq were performed in duplicate for key observations such as H2AK119ub1, H3K27me3, RING1B, SUZ12, CBX7, RYBP and H3K36me2 ChIP-seq.

#### *In situ* HiC

*In situ* HiC was performed as described (Rao et al., 2014) with slight alterations to the protocol. 5x10^6^ ESCs were crosslinked for 10 min in 1% formaldehyde before quenching in 125mM Glycine for 5 min. Cell pellets were washed twice in PBS before contact generation. Pellet was resuspended in 500uL HiC lysis buffer (10mM Tris-HCl pH8.0, 10mM NaCl, 0.2% NP40 and protease inhibitors) and incubated with rotation for 30 min at 4°C. Cells were pelleted and washed in 500uL HiC lysis buffer. Pellet was resuspended in 100uL 0.5% SDS and incubated for 10 min at 62°C. 285uL of H20 and 50uL of 10% Triton X-100 was added to quench the SDS at 37°C for 15 min. 50uL of 10xNEB DpnII buffer and 750U of DpnII were added (NEB R0453M) then incubated at 37°C overnight with shaking. Digestion was heat inactivated the next day for 20 min at 62°C. Biotin fill in was performed by addition of 52uL fill-in mix (0.288mM dCTP/dTTP/dGTP, 50U DNA Polymerase I, Large Klenow fragment (NEB M0210) and 0.288mM biotin-dATP (Thermo 19524016)). Fill-in reaction was incubated at 37°C for 1 h with shaking. Ligation was performed by adding 150uL 10x NEB T4 DNA ligase buffer, 125uL 10% Triton X-100, 3uL 50mg/mL BSA and 8000U of T4 DNA ligase and H20 up to 1.5mL. Ligation was performed overnight at 25°C with shaking. Nuclei were pelleted before resuspending in IP buffer (33 mM Tris-HCl pH 8, 100 mM NaCl, 5 mM EDTA, 0.2% NaN_3_, 0.33% SDS, 1.66% Triton X-100) and sonicating to fragment size between 200-1000bp. 15uL of the sample was de-crosslinked overnight at 65°C with 85ul of decrosslinking buffer (0.1M NaHCO_3_, 1% SDS) before clean up using QiaQuick PCR clean up kit from QIAGEN.

Library prep was performed as described (Mumbach et al., 2016). Streptavidin C-1 beads (Thermo 65001) were washed and resuspended in 2x biotin binding buffer (10mM Tris-HCl pH7.5, 1mM EDTA, 2M NaCl). 10ng of DNA was used for biotin capture, streptavidin C-1 beads were added and incubated for 15 min at room temperature with shaking. Beads were washed twice at 55°C for 2 min in tween wash buffer (5mM Tris-HCl pH7.5, 0.5mM EDTA, 1M NaCl, 0.05% Tween). Beads were washed once in 1xTD buffer (20mM Tris-HCl pH7.5, 10mM MgCl_2_, 20% DMF) before being tagmented with in-house produced Tn5 transposase in TD buffer for 55°C for 10 min. TN5 was quenched in 50mM EDTA for 30 min at 55°C before washing twice in 50mM EDTA, twice in Tween wash buffer and once in 10mM Tris. PCR amplification was carried out on beads using KAPA HotStart Taq enzyme. After purification of PCR product with Ampure beads, the quality of the obtained library was assessed by Bioanalyzer (High Sensitivity DNA kit, Agilent Technologies), prior to sequencing.

#### STORM imaging

For STORM imaging, 35mM MATTEK (P35G-1.5-14-C) plates were gelatinsed with 0.1% gelatin. 500,000 ESC were seeded per plate for 20-22 h before 6 min fixation in 1:1 Methanol: Ethanol at −20°C. Samples were blocked in blocking buffer (PBS with 10% donkey serum) for one h at room temperature. Samples were incubated overnight at 4°C with H3 antibody (AB1791) 1:70 dilution in blocking buffer + 0.1% Triton X-100. Samples were incubated in 1:200 dilution of Alexafluor-647 secondary antibody in PBS in the dark for one h at room temperature. Samples were then post-fixed in 2% PFA for 5 min before storing in PBS at 4°C until imaging.

#### Fractionation and density sedimentation

To obtain nucleosol and chromatin fractions cell pellets from WT+EV, BAP1 KO+EV, BAP1 KO+WT and BAP1 KO+C91S were resuspended in Hypotonic buffer (20mM HEPES pH 7.5, 0.5% Triton X-100, 50mM NaCl, 3mM MgCl2, 300 mM Sucrose, 2 μg/mL Aprotinin, 1 μg/mL Leupeptin, 1mM PMSF) and incubated at 4°C for 15 min. Then 0.3% of Triton X-100 was added to the solution and vortexed for 30 s followed by clarification at 13,000 RPM in a 4°C centrifuge for 10 min The lysate suspension was transferred to a new tube and labeled “Nucleosol fraction..” The insoluble pellet was washed once with hypotonic buffer before resuspension in S150 buffer (20 mM Tris-HCl pH 8.0,150 mM NaCl, 10% glycerol, 0.2% NP40) complemented with Benzonase (25U/μL Millipore) for 1 h at 4°C on rotation. The solution was clarified at 13,000 RPM in a 4°C centrifuge for 10 min. The lysate suspension was transferred to a new tube and labeled “Chromatin fraction.”

For density sedimentation analysis nucleosol and chromatin fractions were loaded onto a linear 10 mL 5%–40% glycerol gradient containing 25mM HEPES pH 7.9, 0.1 mM EDTA, 12.5 mM MgCl2, 100mM KCl, prepared in a centrifuge tube (355603, Beckman). Tubes were centrifuge in a SW40 rotor at 4 degree for 48 h at 40000 rpm. 500ul fractions were manually collected from the top of the gradient and concentrated using 20ul of Strataclean beads (400714, Agilent). Beads were directly resuspended in Laemmli reduced buffer and then used for western blot analysis.

#### Mass spectrometry

For FLAG-IP mass spectrometry, co-immunoprecipitations were performed on 1 mg of nucleosol and chromatin extracts of BAP1+WT, BAP1 KO+EV, BAP1 KO+C91S lysates using M2 agarose beads (30 μL slurry for IP, A2220 Anti-FLAG M2 affinity gel) for 2 h at 4 degrees while rotating. Immunocomplexes were washed with S300 buffer and eluted by competition with 3x Flag peptide (500 ng/ul; SIGMA) twice for 30 min at 16°C and then resuspended in Laemmli sample buffer. Protein lysates were separated on SDS-PAGE gels and processed for mass spectrometry analysis. Proteins from BAP1+WT, BAP1 KO+EV, BAP1 KO+C91S purifications were separated for 2 cm by SDS–PAGE, using 4%–12% NuPAGE Novex Bis–Tris gels (Invitrogen)and NuPAGE MES SDS running buffer (Invitrogen) and then stained with Coomassie Blue using InstantBlue Comassie (Expedeon). A single gel band for each sample was cut and digested with trypsin (Promega) and incubated overnight at 37°C for protein digestion. Then, peptide extraction was carried out and the resulting peptide mixture were combined, desalted and concentrated using StageTip (Proxeon Biosystems) columns, washed with 30 μL of 0.1% Formic acid (FA) and finally eluted with 40 μL of 80% MeCN in 0.1% FA. The samples were concentrated in a vacuum concentrator (Eppendorf concentrator 5301) and peptides were dissolved in 7 μL of 0.1% FA. Approximately 6 μL of purified peptide mixture were analyzed on a LC–ESI–MS-MS Q-Exactive HF hybrid quadrupole-Orbitrap mass spectrometer (Thermo Fisher Scientific), using a gradient of 80 min with a flow of 250 nL/min. Full scan MS spectra were acquired in a range of m/z 300–1650.

For mass spectrometry of chromatin bound proteins sub-cellular fractionation into cytosol, nucleosol and chromatin compartments, cell lysates were obtained according to established protocols (Méndez and Stillman, 2000). Briefly, to prepare nuclear extracts, the cells were washed twice with PBS and once with hypotonic buffer. Then, cells were incubated with hypotonic buffer (20 mM HEPES pH 8.0, 5 mM KCl, 1.5 mM MgCl_2_, 0.1 mM dithiothreitol [DTT]) for 30 min on ice. Triton X-100 (0.1%) was added, and the cells were incubated for 5 min on ice. Nuclei were collected in pellet by centrifugation (2 min, 3750 × *rpm*, 4°C). The supernatant was discarded and nuclei were washed once in hypotonic buffer, and then lysed in Urea buffer (8M Urea in 150 TrisHCl,pH 7.6, protease inhibitors). After 30 min on ice, insoluble proteins were removed from the nuclear extract by high-speed centrifugation (30 min, 13000 × *g*, 4°C). Protein extracts in UREA buffer were lysate, reduced, alkylated, digested and cleaned using the iST kit (Preomics).

### Quantification and statistical analysis

#### ChIP-seq data analysis

Paired-end DNA reads were processed through fastp to trim adapters and to remove low quality nucleotides at read ends (Chen et al., 2018). Quality-filtered DNA reads were aligned to the mouse reference genome mm10, or mm10 and fly reference genome (dm6) for histone ChIP-Rx using Bowtie v1.2.2 retaining only uniquely aligned reads (-m 1) and using the parameters -I 10, -X 1000 (Langmead et al., 2009). Reads mapped to both mm10 and dm6 were discarded. Peaks were identified using MACS2 v2.1.1 in narrow mode with parameters –format BAMPE –keep-dup all -m 3 30 and p value 1e-10 (Zhang et al., 2008). Peaks were annotated using the R package ChIPpeakAnno v3.15 using for each peak the 5 kbp region around the center of the peak (Zhu et al., 2010).

The intensity of histone modifications or transcription factor binding was represented through boxplots or heatmaps, both generated from BigWig files that were obtained using the function bamCompare from deepTools 3.1 (Ramírez et al., 2016) with parameters –binSize 50 –extendReads. The –scaleFactors parameter of bamCompare was set to (1/total mapped reads)^∗^1,000,000 to normalize for the differences in sample library size. Samples obtained with the ChIP-Rx method, were normalized using (1/dm mapped reads)^∗^1,000,000 as –scaleFactors parameter (Orlando et al., 2014). ChIP-Rx samples were also normalized by the mm10/dm6 mapped reads of the input samples. A single genomic DNA input was sequenced for each cell line and used for the normalization of the respective ChIP-Rx samples. The preparation of the heatmaps required the generation of a data matrix through computeMatrix with parameters –referencePoint TSS/center -a 5000 -b 5000 (Ramírez et al., 2016). The data matrix was converted into heatmap by plotHeatmap (Ramírez et al., 2016). Boxplots were prepared using multiBigwigSummary in BED-file mode and using as bed file the regions corresponding to promoters (TSS ± 2.5 kbp) or distal genomic regions such as enhancers (Ramírez et al., 2016). P values and effect sizes displayed in boxplot quantifications of ChIP-seq data were calculated using non-parametric rank tests. For p values, Mann-Whitney’s U Test was performed, meanwhile for the effect sizes we applied the rank-biserial correlation. These calculations were done using the r functions wilcox.test (r-base) and rank_biserial (effectsize package) (Sullivan and Feinn, 2012), respectively.

Density plots were prepared using as input the intensity of histone modifications on 5 kbp windows at genome-wide level. When the log2 ratio between two conditions was reported, “Inf” and “-Inf” values were equaled to the maximum and minimum finite values, respectively.

Venn diagram were prepared by the R package VennDiagram v1.6.20 using as input the name of the genes targeted by a specific protein or histone modification (Chen and Boutros, 2011). Intergenic sites were defined by subtracting from the mouse genome the region included between the transcription starting site and transcription end site of each gene. Sequences annotated as MERVL, IAPEz and LINE were filtered from the RepeatMasker annotation of the repeated sequences of the mouse genome using RepeatMasker-open 4.0 (AFA. Smit, R. Hubley). The histone modifications were quantified on the whole length of each of these transposable elements by multiBigwigSummary in BED-file mode.

#### RNA-seq analysis

RNA-seq was performed following SMART-seq2 protocol (Picelli et al., 2014) with minor modifications. Briefly, poly-A containing mRNA molecules obtained from 1 μg of total RNA were copied into first-strand cDNA by reverse transcription and template-switching using oligo (dT) primers and an LNA-containing template-switching oligo (TSO). Resulting cDNA was pre-amplified with KAPA HotStart Taq enzyme (Kapa Biosystems) and then purified with Ampure beads (Agencourt AMPure XP- Beckman Coulter). One nanogram of pre-amplified cDNA was tagmented with in-house produced Tn5 transposase and further amplified with KAPA HotStart Taq enzyme. After purification with Ampure beads, the quality of the obtained library was assessed by Bioanalyzer (High Sensitivity DNA kit, Agilent Technologies), prior to sequencing. Each RNA-seq experiment was performed in triplicate.

RNA reads were aligned to the mouse reference genome mm10 using STAR v2.7 without allowing for multimapping. PCR duplicates were removed using samblaster (Faust and Hall, 2014). Mapped reads were assigned to genes using featureCounts (Liao et al., 2014) with unstranded read counting on exons and using the gene name as attribute type in the annotation. Genes were annotated as in Gencode M21 (GRCm38) downloaded from https://www.gencodegenes.org/mouse/. Differentially expressed genes were identified using the R package DESeq2 v1.24 using default parameters (Love et al., 2014). The fold change of lowly expressed genes was corrected using the lfcShrink function of the apeglm R package with the type option set to apeglm v1.6 (Zhu et al., 2019). The adjusted p value was corrected by the independent hypothesis weighting (IHW) method as implemented in the R package IHW v1.12 (Ignatiadis et al., 2016). Genes were considered differentially expressed when presenting an absolute log2 fold change equal or greater than 1 and an adjusted p value lower than 0.05.

#### HiC data analysis

DNA reads were filtered for low-quality bases and adapters using fastp (Chen et al., 2018). Filtered DNA reads were processed through the HiCpro pipeline to obtain contact matrices reporting the chromatin interactions at the genome-wide level (Servant et al., 2015). Using default settings, DNA reads were aligned to the mouse reference genome mm10 using bowtie2 in end-to-end mode, uniquely aligned DNA reads were assigned to the DpnII genomic restriction fragments, valid interactions were identified and used to generate interaction matrices. The HiCpro matrices containing all of the valid pairs were converted in *hic* format using the hicpro2juicebox function (Robinson et al., 2018), and in *cool* format using the hic2cool function (https://github.com/4dn-dcic/hic2cool). Contact matrices in cool format where corrected using the ICE algorithm as implemented in the hicCorrectMatrix function (Wolff et al., 2020). Plots of the interactions occurring at single locus or whole-chromosome level were obtained by HiCplotter (Akdemir and Chin, 2015). HiC was performed in duplicate with key findings being overserved in each before pooling replicates to make the final high-depth analyses.

#### STORM and DAPI analysis

Direct STORM (dSTORM) imaging was performed using the Nikon N-STORM microscope equipped with a 1.49 NA CFI Apochromat TIRF objective, exciting the Alexa Fluor 647 dye with the 647 nm laser light in HILO (highly inclined and laminated optical sheet) mode (Tokunaga et al., 2008). The 405 nm laser light (activation laser) was used for reactivating the Alexa Fluor 647 into a fluorescent state. The activation laser power was automatically increased by the NIS software to keep the number of localizations per frame constant up to a maximum of 50% of the laser power. Each dSTORM acquisition consisted of 40 thousand images recorded with an Orca-Flash4.0 sCMOS camera (Hamamatsu) with an exposure time of 20 ms, a pixel size of 161.5 nm and a field of view of 128x128 pixels. During dSTORM acquisitions, cells were kept in imaging buffer (100 mM MEA, 1% glucose, 560 ug/mL Glucose Oxidase and 34 ug/mL Catalase in PBS).

Two regions of interest (ROI) of 32x32 pixels for each dSTORM image were processed using ThunderSTORM (Ovesný et al., 2014) with a pre-detection wavelet filter (B-spline, scale 2, order 3), initial detection by non-maximum suppression (radius 1, threshold at one standard deviation of the F1 wavelet), and sub-pixel localization by integrated Gaussian point-spread function and maximum likelihood estimator with a fitting radius of 3 pixels. Detected localizations were filtered (intensity > 500 photons, sigma range of 50–500, and localization uncertainty < 20 nm). The filtered dataset was then corrected for sample drift (cross-correlation of images from five bins at a magnification of 5) and repeated localizations was removed by merging points which reappeared within 20 nm. STORM images were visualized using the normalized Gaussian method with a lateral uncertainty of 20 nm.

Cluster analysis were performed thanks to a supervised machine learning approach using trained neural network (Williamson et al., 2020). The CAML (Cluster Analysis by Machine Learning) analysis workflow consisting of 3 stages and corresponding Python scripts, was used to: 1) prepare the data converting the x,y localization tables in a list of near-neighbor distances; 2) evaluate the input data with a trained model; 3) extract clustering information. The 87B144 model was used (Williamson et al., 2020), considering the possibility that more than 100 localizations per cluster could occur in our dataset.

After STORM acquisitions, cells were stained with DAPI 10 um/mL in PBS for 30 min, washed and imaged with a 100x 1.45 NA oil immersion objective lens on a CSU-W1 Yokogawa Spinning Disk confocal system with a 50 um pinhole disk mounted on a Nikon Eclipse Ti2 stative and equipped with a motorized xyz stage, 6 solid state lasers and a Prime BSI sCMOS camera. More than 40 fields of view (FOV) per condition and a 20um Z stack with a voxel size of 65x65x200 nm were acquired. The nuclei area was quantified using a custom-made ImageJ macro. Briefly, the H3-Alexa647 and the DAPI signals were added to get a more homogeneous fluorescence signal in the nucleus, then, a Z maximum projection was made, and the nuclei segmented with the Otsu algorithm after a median filter of 5 pixels was applied. The analyze particles function of the ImageJ software was applied and each field of view was then manually checked to exclude from the analysis the not correctly segmented nuclei. More than 100 nuclei were analyzed for each condition.

#### Mass spectrometry analysis

For mass spectrometry data analysis, the acquired tandem mass spectra were searched against the Uniprot mouse database using MaxQuant (version 1.6.2.3) (Tyanova et al., 2016a). Trypsin was specified as a cleavage enzyme, allowing up to three missed cleavages. Carbamidomethylation of cysteine was used as a fixed modification, with oxidation of methionine used as variable modifications. For label-free quantification the “match between runs” option in MaxQuant was applied. A false discovery rate of 1% was applied to peptide and protein identifications. Contaminants and reverse hits were excluded from the results prior to further analysis. Volcano plots were created using Perseus (version 1.6.2.3) (Tyanova et al., 2016b). Volcano plots and stoichiometric analysis were performed as described (Smits et al., 2013). Plots were generated using GraphPad PRISM version 8.4.3.

#### Statistics

Sample size and statistical tests are indicated in STAR methods, and/ or in the figure legends. For ChIP-seq analyses individual biological replicates (n = 2) are both shown in the main and supplemental figures. RNA-seq were performed in biological triplicate (n = 3). HiC was performed in biological duplicate (n = 2), with key findings being observed in both replicates before pooling data for higher resolution and depth of contact maps. Individual replicate information for HiC is present in the supplemental figures. All replicates were obtained by measuring distinct samples (biological and/or experimental replicates) and not by measuring multiple times the same sample (technical replicates). No methods were used to test assumptions of the statistical approach.

## Supplemental information

Document S1. Figures S1–S5

Table S1. ChIP-seq information, related to Figures 1, 2, 3, 4, 5, and 7 Table containing information on number of peaks per ChIP-seq experiment.

Table S2. List and details of DEGs, related to Figure 1 Table containing details on DEGs, including gene list, gene ontology, and a list of PcG protein target genes.

Table S3. gRNA and qPCR primer sequences, related to Figures 1, 3, 5, and 7 Table containing information on gRNA and qPCR primer sequences used in this study. and Tables S1 and S2

Document S2. Article plus supplemental information
