# Supplementary material for: BAP1 enhances Polycomb repression by counteracting widespread H2AK119ub1 deposition and chromatin condensation
Source: Mol Cell. 2021 Sep 2;81(17):3526–3541.e8. doi: 10.1016/j.molcel.2021.06.020 (PMC8428331; doi:10.1016/j.molcel.2021.06.020)
Supplement: Document S1. Figures S1–S5 [file mmc1.pdf]

Molecular Cell, Volume *81*

Supplemental information

BAP1 enhances Polycomb repression by counteracting widespread H2AK119ub1 deposition and chromatin condensation

Eric Conway, Federico Rossi, Daniel Fernandez-Perez, Eleonora Ponzo, Karin Johanna Ferrari, Marika Zanotti, Daria Manganaro, Simona Rodighiero, Simone Tamburri, and Diego Pasini

Figure S1

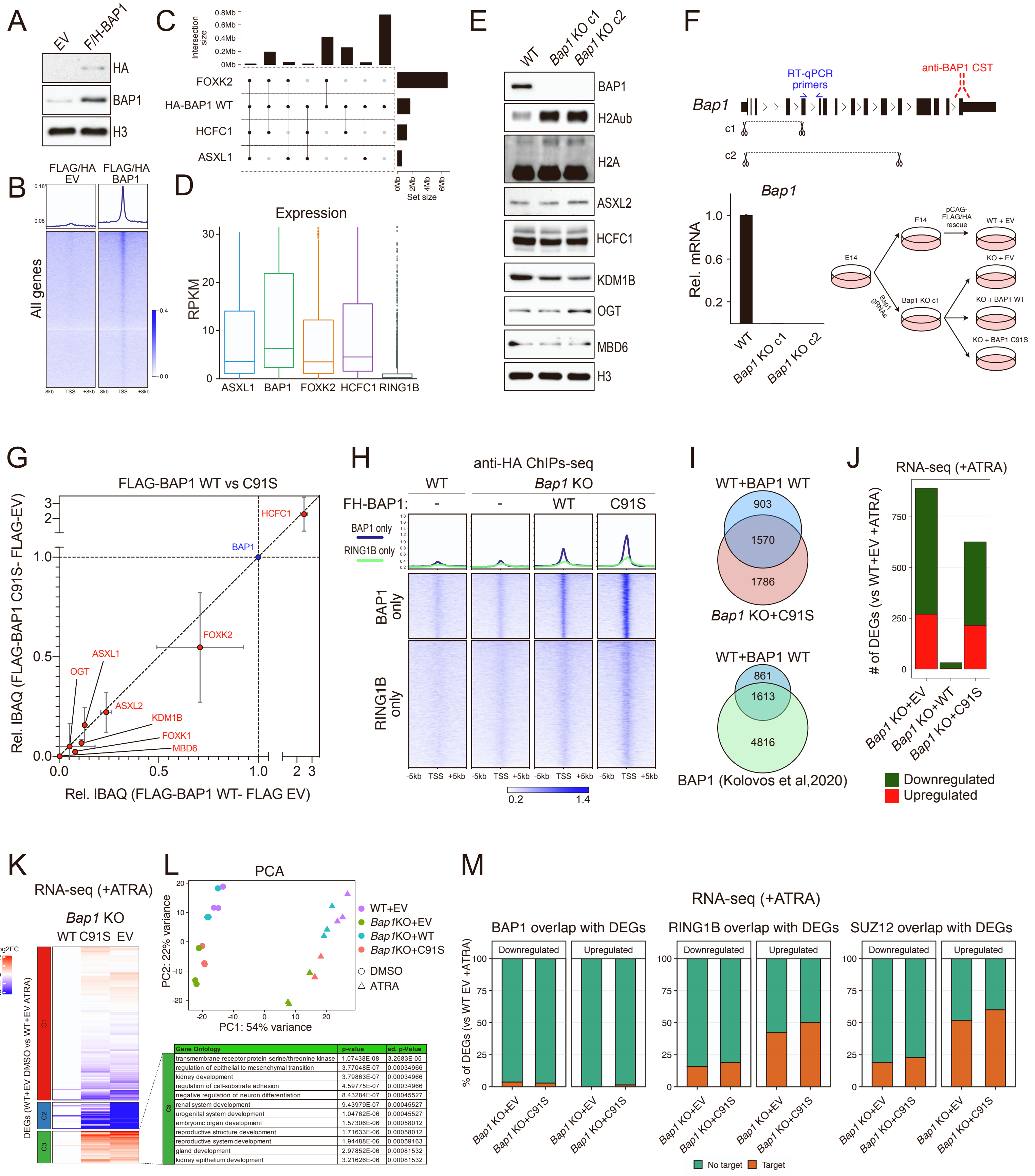

Gene Ontology	p-value	ad. p-Value
transmembrane receptor protein serine/threonine kinase	1.07438E-08	3.2683E-05
regulation of epithelial to mesenchymal transition	3.77048E-07	0.00034966
kidney development	3.79863E-07	0.00034966
regulation of cell-substrate adhesion	4.59775E-07	0.00034966
negative regulation of neuron differentiation	8.43284E-07	0.00045527
renal system development	9.43979E-07	0.00045527
urogenital system development	1.04762E-06	0.00045527
embryonic organ development	1.57306E-06	0.00058012
reproductive structure development	1.71633E-06	0.00058012
reproductive system development	1.94488E-06	0.00059163
gland development	2.97852E-06	0.00081532
kidney epithelium development	3.21626E-06	0.00081532

Figure S1. Supporting material for figure 1

A) Western blot analysis with the indicated antibodies on total protein extracts from the indicated ESC cell lines (E14 WT + empty vector, E14 WT + FH-BAP1 WT).

B) Heatmaps representing normalized ChIP-seq intensity for HA in the E14 WT + empty vector and E14 WT + FH-BAP1 WT cell lines +/- 8kb of TSS. All TSS are shown.

C) Upset plot for peak coverage from the indicated ChIP-seq showing overlap between the different PR-DUB subunit ChIPs.

D) Boxplots showing the expression levels obtained from RNA-seq analyses in WT mESCs for the target genes of the indicated PR-DUB or PRC1 proteins.

E) Western blot analysis with the indicated antibodies on total protein extracts from the indicated ESC cell lines.

F) BAP1 KO strategy. Top: position of gRNA pairs, qRT-PCR primers and BAP1 antibody epitope relative to the BAP1 gene locus. Bottom left: qRT-PCR of BAP1 in cDNA from the indicated cell lines. Data are represented as mean ±SD. Bottom right: strategy for generating BAP1 WT and catalytic mutant rescue cell lines.

G) Comparison of stoichiometry (IBAQ relative to BAP1 WT or C91S mutant) of PR-DUB subunits in FLAG/HA-BAP1 IP mass spectrometry purifications from nucleosol lysates. Data are represented as mean ±SD.

H) Metaplots and heatmaps representing ChIP-seq intensity for HA in the indicated cell lines +/- 5kb of TSS. Clusters are divided into BAP1 target genes only and RING1B only targets.

I) Venn diagram overlap of called peaks from the indicated ChIP-seq.

J) Bar chart showing number of differentially expressed genes (DEGs) in the indicated cell lines (all +ATRA) relative to WT+EV +ATRA.

K) Left: RNA-seq heatmap of those genes differentially expressed in WT+EV DMSO vs WT+EV +ATRA. Clusters are separated into those not changed (C1), downregulated (C2), and upregulated (C3) in *Bap1* KO+EV +ATRA. Right: Top ranking gene ontology terms for C3 genes.

L) Principal Component Analysis (PCA) of RNA-seq triplicates for the indicated cell lines in both DMSO and ATRA treated conditions.

M) Percentage overlap of differentially expressed genes (DEG) from S1J with either HA-BAP1, RING1B or SUZ12 ChIP-seq targets.

Figure S2

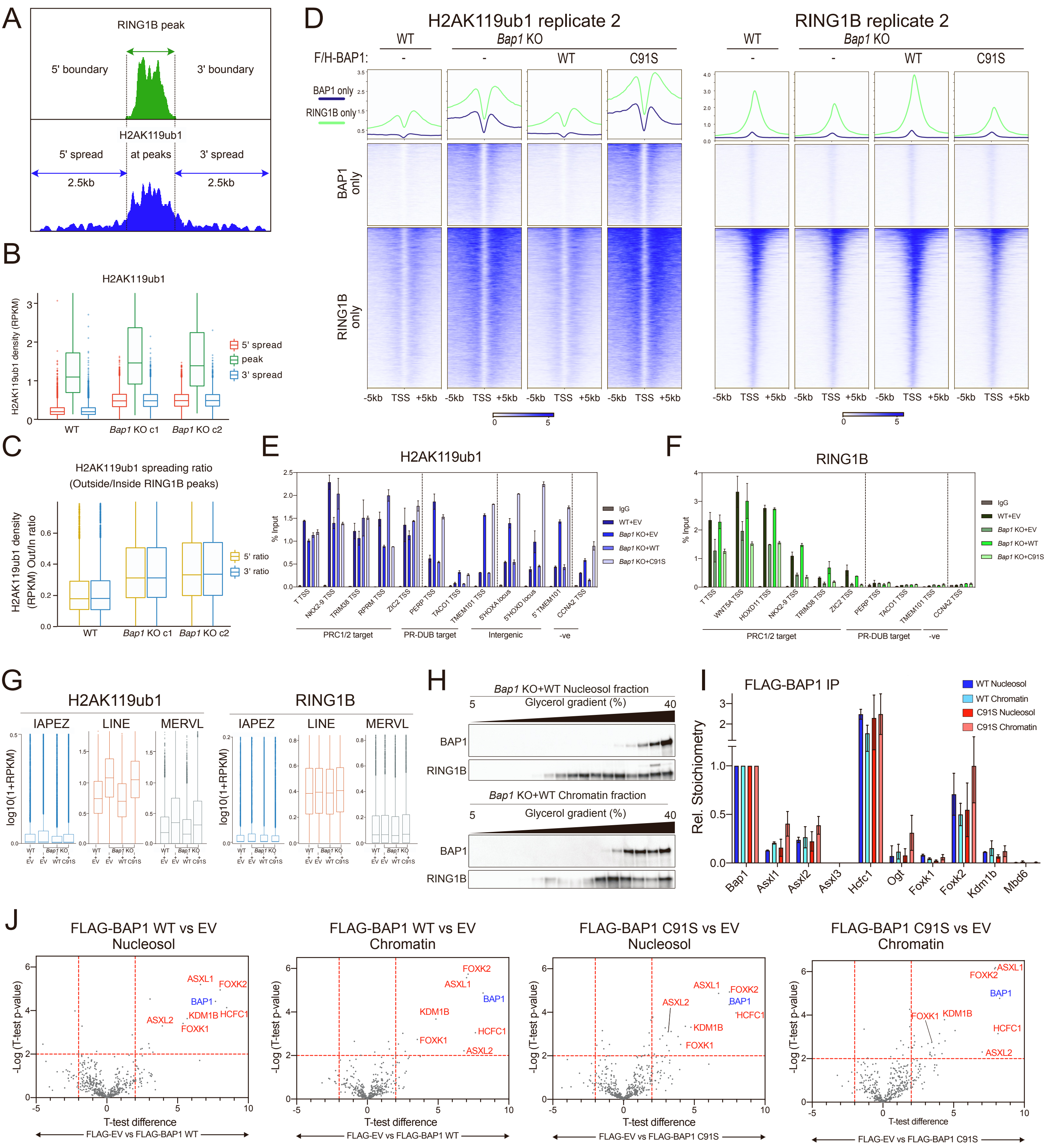

A
RING1B peak
5' boundary
3' boundary
H2AK119ub1
5' spread
at peaks
3' spread
2.5kb
2.5kb
B
H2AK119ub1
H2AK119ub1 density (RPKM)
5' spread
peak
3' spread
WT
Bap1 KO c1
Bap1 KO c2
C
H2AK119ub1 spreading ratio
(Outside/Inside RING1B peaks)
H2AK119ub1 density (RPKM) Out/In ratio
5' ratio
3' ratio
WT
Bap1 KO c1
Bap1 KO c2
D
H2AK119ub1 replicate 2
RING1B replicate 2
WT
Bap1 KO
F/H-BAP1:
-
WT
C91S
BAP1 only
RING1B only
-5kb TSS +5kb
E
H2AK119ub1
% Input
IgG
WT+EV
Bap1 KO+EV
Bap1 KO+WT
Bap1 KO+C91S
T TSS
NKX2-9 TSS
TRIM38 TSS
RPRM TSS
ZIC2 TSS
PERP TSS
TACO1 TSS
TMEM101 TSS
5'HOXA locus
5'HOXD locus
5' TMEM101
CCNA2 TSS
PRC1/2 target
PR-DUB target
Intergenic
-ve
F
RING1B
% Input
IgG
WT+EV
Bap1 KO+EV
Bap1 KO+WT
Bap1 KO+C91S
T TSS
WNT5A TSS
HOXD11 TSS
NKX2-9 TSS
TRIM38 TSS
ZIC2 TSS
PERP TSS
TACO1 TSS
TMEM101 TSS
CCNA2 TSS
PRC1/2 target
PR-DUB target
-ve
G
H2AK119ub1
RING1B
IAPEZ
LINE
MERVL
log10(1+RPKM)
WT EV
Bap1 KO EV WT C91S
H
Bap1 KO+WT Nucleosol fraction
Glycerol gradient (%)
5
40
BAP1
RING1B
Bap1 KO+WT Chromatin fraction
I
FLAG-BAP1 IP
Rel. Stoichiometry
WT Nucleosol
WT Chromatin
C91S Nucleosol
C91S Chromatin
Bap1
Asxl1
Asxl2
Asxl3
Hcfc1
Ogt
Foxk1
Foxk2
Kdm1b
Mbd6
J
FLAG-BAP1 WT vs EV
Nucleosol
Chromatin
FLAG-BAP1 C91S vs EV
-Log (T-test p-value)
T-test difference
FLAG-EV vs FLAG-BAP1 WT
FLAG-EV vs FLAG-BAP1 C91S
ASXL1
FOXK2
BAP1
KDM1B
HCFC1
ASXL2
FOXK1

Figure S2. Supporting material for figure 2

A) Illustration describing criteria used to select RING1B inside peak regions, its boundaries, and the regions selected for the analysis at the 5' and 3' outside of RING1B peak boundaries.

B) Boxplots representing H2AK119ub1 density distributions in WT and *Bap1* KO cells (two clones) within RING1B peaks, as well as 2.5kb outside of the 5' and 3' ends.

C) Boxplots representing the distribution of the H2AK119ub1 density ratio between the H2AK119ub1 density inside RING1B peaks and at 5' or 3' spreading regions.

D) Metaplots and heatmaps representing normalized ChIP-seq intensity for second replicates of H2AK119ub1 or RING1B in the indicated cell lines +/- 5kb of TSS. Clusters are divided into BAP1 target genes only and RING1B only targets.

E) ChIP-qPCR validation of H2AK119ub1 ChIP results at the indicated PRC1/2 or PR-DUB targets and intergenic sites. Data are represented as mean ±SD.

F) ChIP-qPCR validation of RING1B ChIP results at the indicated PRC1/2 or PR-DUB targets. Data are represented as mean ±SD.

G) Boxplots representing H2AK119ub1 (left) or RING1B (right) ChIP-seq log10(1+RPKM) levels in the indicated cell lines at the indicated types of intergenic repeats.

H) Glycerol gradient on nucleosol or chromatin extracts from *Bap1* KO+WT cells followed by Western blot using the indicated antibodies.

I) Bar chart of stoichiometry (IBAQ relative to BAP1) of PR-DUB subunits in FLAG/HA-BAP1 IP mass spectrometry purifications from nucleosol or chromatin lysates for either BAP1 WT or C91S. Data are represented as mean ±SD.

J) Volcano plot of FLAG/HA-BAP1 IP mass spectrometry data (either WT or C91S mutant in both nucleosol and chromatin) plotting T-test difference of LFQ values on the X-axis against -Log (T-test p-value). Significantly interacting PR-DUB subunits are labelled in red.

Figure S3

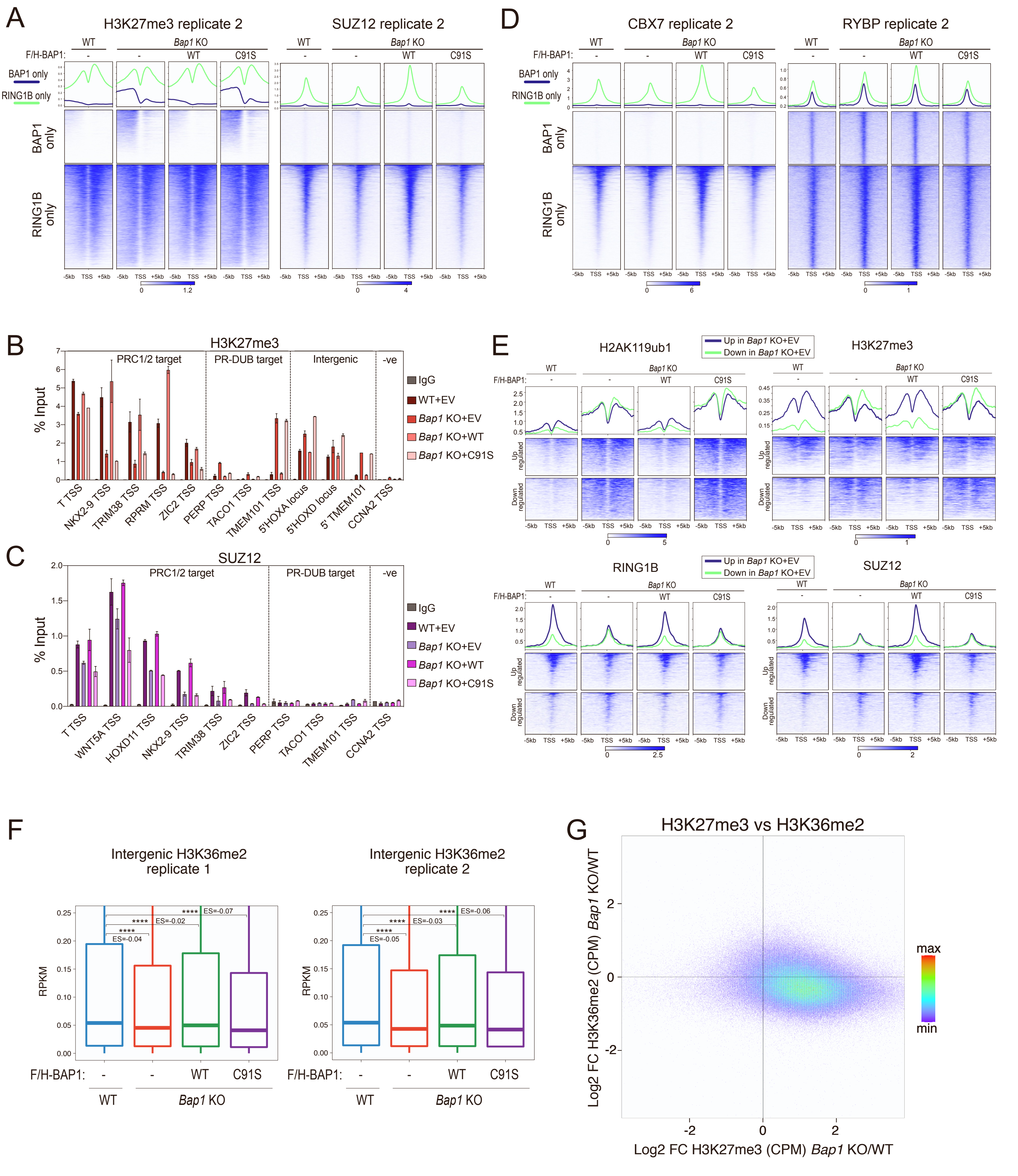

A
H3K27me3 replicate 2
SUZ12 replicate 2
WT
Bap1 KO
F/H-BAP1:
C91S
BAP1 only
RING1B only
-5kb TSS +5kb
0 1.2
0 4
B
H3K27me3
PRC1/2 target
PR-DUB target
Intergenic
-ve
% Input
IgG
WT+EV
Bap1 KO+EV
Bap1 KO+WT
Bap1 KO+C91S
T TSS
NKX2-9 TSS
TRIM38 TSS
RPRM TSS
ZIC2 TSS
PERP TSS
TACO1 TSS
TMEM101 TSS
5'HOXA locus
5'HOXD locus
5' TMEM101
CCNA2 TSS
C
SUZ12
WNT5A TSS
HOXD11 TSS
D
CBX7 replicate 2
RYBP replicate 2
0 6
0 1
E
H2AK119ub1
H3K27me3
Up in Bap1 KO+EV
Down in Bap1 KO+EV
Up regulated
Down regulated
0 5
RING1B
SUZ12
0 2.5
0 2
F
Intergenic H3K36me2 replicate 1
Intergenic H3K36me2 replicate 2
RPKM
**** ES=-0.04
**** ES=-0.02
**** ES=-0.07
**** ES=-0.05
**** ES=-0.03
**** ES=-0.06
G
H3K27me3 vs H3K36me2
Log2 FC H3K36me2 (CPM) Bap1 KO/WT
Log2 FC H3K27me3 (CPM) Bap1 KO/WT
max
min

Figure S3. Supporting material for figure 4

A) Metaplots and heatmaps representing normalized ChIP-seq intensity for second replicates of H3K27me3 or SUZ12 in the indicated cell lines +/- 5kb of TSS. Clusters are divided into BAP1 target genes only and RING1B only targets.

B) ChIP-qPCR validation of H3K27me3 ChIP results at the indicated PRC1/2 or PR-DUB targets and intergenic sites. Data are represented as mean ±SD.

C) ChIP-qPCR validation of SUZ12 ChIP results at the indicated PRC1/2 or PR-DUB targets. Data are represented as mean ±SD.

D) Metaplots and heatmaps representing normalized ChIP-seq intensity for second replicate of CBX7 (left) and RYBP (right) in the indicated cell lines +/- 5kb of TSS. Clusters are divided into BAP1 target genes only and RING1B only targets.

E) Metaplots and heatmaps representing normalized ChIP-seq intensity for the indicated antibodies, in the indicated cell lines +/- 5kb of TSS at genes that are differentially expressed in *Bap1* KO+EV vs WT+EV ESC.

F) Boxplots representing two replicates of H3K36me2 ChIP-seq RPKM levels in the indicated cell lines at intergenic sites (n=38,068).

G) Genome wide comparison of ChIP-seq signal using 5kb windows. Log2 fold change H3K27me3 ChIP-seq for *Bap1* KO+EV vs WT+EV comparison (X-axis) plotted against Log2 fold change of H3K36me2 ChIP-seq (Y-axis). Each dot represents one 5kb window.

Figure S4

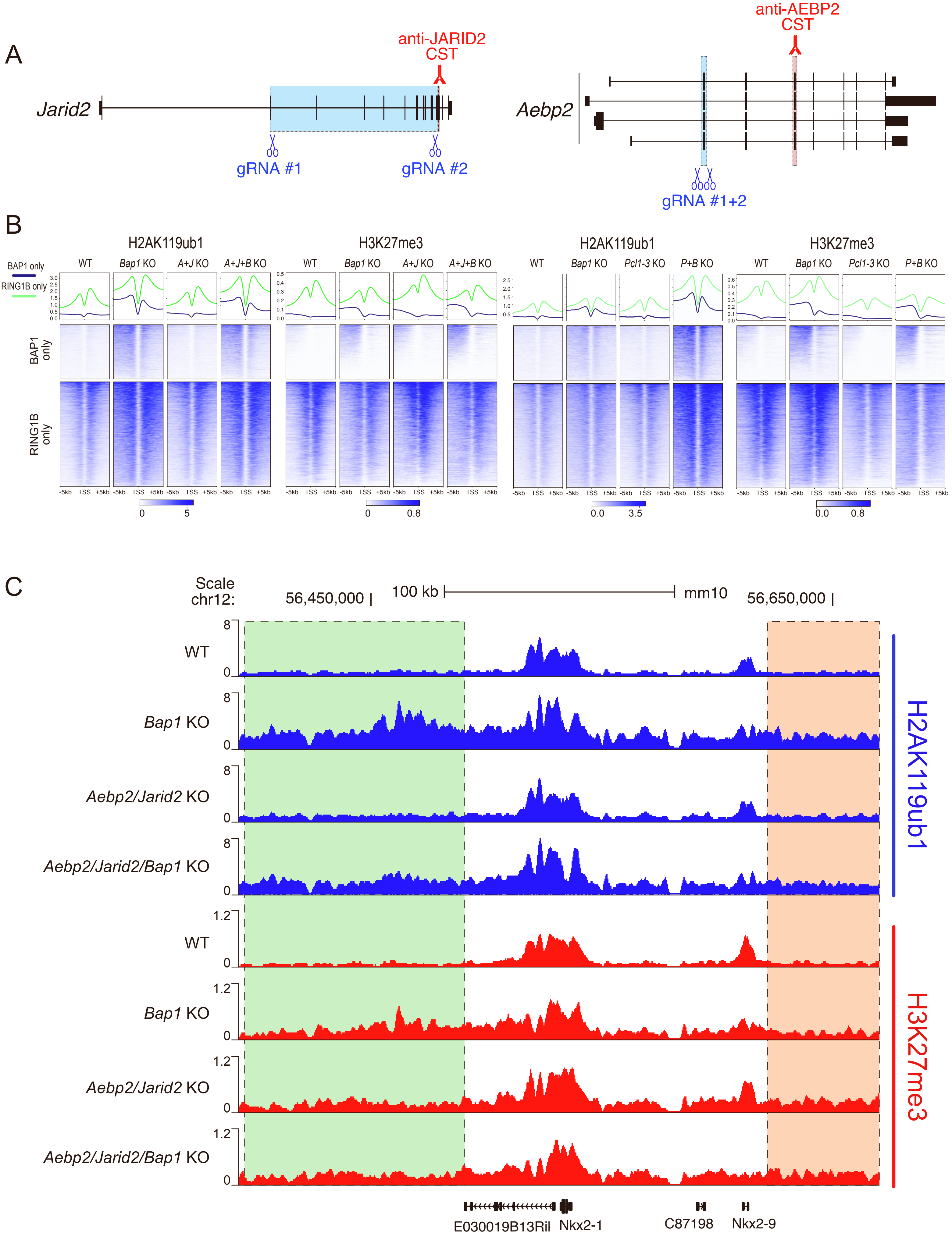
A
Jarid2
anti-JARID2 CST
gRNA #1
gRNA #2
Aebp2
anti-AEBP2 CST
gRNA #1+2
B
H2AK119ub1
H3K27me3
BAP1 only
RING1B only
WT
Bap1 KO
A+J KO
A+J+B KO
Pcl1-3 KO
P+B KO
-5kb TSS +5kb
C
Scale
chr12:
56,450,000
100 kb
mm10
56,650,000
Aebp2/Jarid2 KO
Aebp2/Jarid2/Bap1 KO
H2AK119ub1
H3K27me3
E030019B13Ril
Nkx2-1
C87198
Nkx2-9

Figure S4. Supporting material for figure 5

A) *Jarid2* (left) and *Aebp2* (right) KO strategy. Position of gRNA pairs and antibody epitope relative to the gene locus is shown.

B) Metaplots and heatmaps representing normalized ChIP-seq intensity for H2AK119ub1 and H3K27me3 in the indicated cell lines +/- 5kb of TSS. Clusters are divided into BAP1 target genes only and RING1B only targets.

C) UCSC genome browser snapshot of H2AK119ub1 and H3K27me3 ChIP-seq in the indicated cell lines.

Figure S5

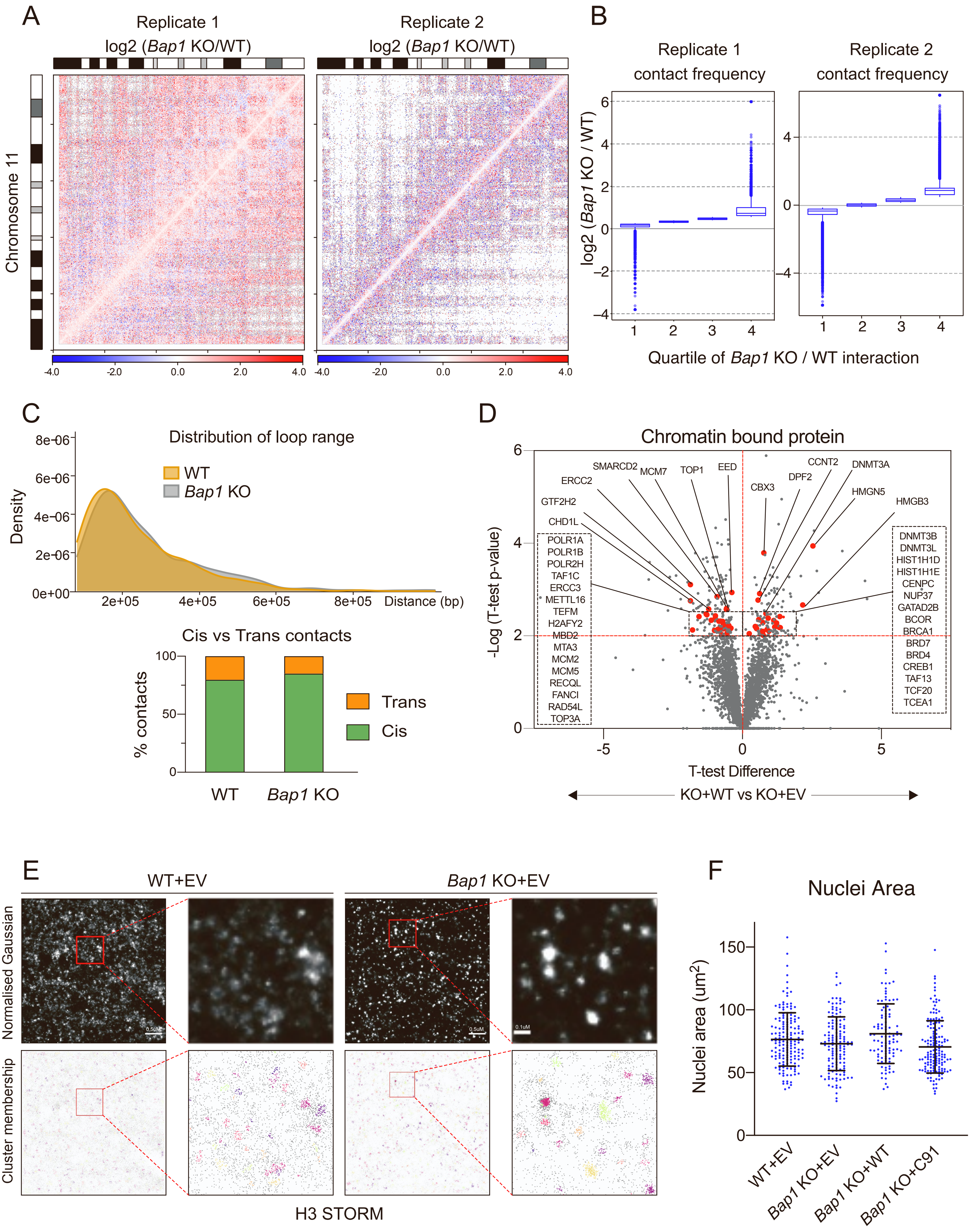

A
Replicate 1
log2 (Bap1 KO/WT)
Replicate 2
log2 (Bap1 KO/WT)
Chromosome 11
-4.0 -2.0 0.0 2.0 4.0
B
Replicate 1
contact frequency
Replicate 2
contact frequency
log2 (Bap1 KO / WT)
Quartile of Bap1 KO / WT interaction
C
Distribution of loop range
WT
Bap1 KO
Density
Distance (bp)
Cis vs Trans contacts
% contacts
Trans
Cis
WT
Bap1 KO
D
Chromatin bound protein
-Log (T-test p-value)
T-test Difference
KO+WT vs KO+EV
SMARCD2
MCM7
TOP1
EED
CCNT2
DNMT3A
ERCC2
DPF2
CBX3
GTF2H2
HMGN5
HMGB3
CHD1L
POLR1A
POLR1B
POLR2H
TAF1C
ERCC3
METTL16
TEFM
H2AFY2
MBD2
MTA3
MCM2
MCM5
RECQL
FANCI
RAD54L
TOP3A
DNMT3B
DNMT3L
HIST1H1D
HIST1H1E
CENPC
NUP37
GATAD2B
BCOR
BRCA1
BRD7
BRD4
CREB1
TAF13
TCF20
TCEA1
E
WT+EV
Bap1 KO+EV
Normalised Gaussian
Cluster membership
H3 STORM
F
Nuclei Area
Nuclei area (um2)
WT+EV
Bap1 KO+EV
Bap1 KO+WT
Bap1 KO+C91

Figure S5. Supporting material for figure 6

A) Ice-normalised HiC contact matrix of the entire chromosome 11 in log2 (Fold change BAP1 KO/WT) at 250kbp resolution in each of the two replicates performed.

B) Boxplot of contact frequency of log2(BAP1 KO/WT) ratios divided into quartiles in individual replicates.

C) Top: Plot of contact frequency vs distance for HiC valid contacts. Bottom: Bar chart showing percentages of Cis/Trans contacts from the HiC data.

D) Volcano plot of mass spectrometric analysis of chromatin bound proteins in *Bap1* KO+BAP1 WT vs *Bap1* KO+EV. pValue=0.001

E) Representative STORM images of WT+EV and *Bap1* KO+EV ESC stained with Histone H3 antibody. Both normalised gaussian and cluster membership images are shown for the same regions of interest. Scale bars of 0.5μm (left column) and 0.1μm (right column) are shown.

F) Quantification of DAPI stained nuclear area in multiple fields of view in the indicated cell lines. Data are represented as mean ±SD.

Table S1. ChIP-seq information, related to Figure 1-5 and 7.

Table containing information on number of peaks per ChIP-seq experiment.

Table S2. List and details of DEGs, related to Figure 1.

Table containing details on DEGs, including gene list, gene ontology and a list of PcG protein target genes.

Table S3. gRNA and qPCR primer sequences, related to Figure 1,3,5 and 7.

Table containing information on gRNA and qPCR primer sequences used in this study.
